# A Smartphone App Designed to Help Cancer Patients Stop Smoking: Results From a Pilot Randomized Trial on Feasibility, Acceptability, and Effectiveness

**DOI:** 10.2196/16652

**Published:** 2020-01-17

**Authors:** Jonathan B Bricker, Noreen L Watson, Jaimee L Heffner, Brianna Sullivan, Kristin Mull, Diana Kwon, Johann Lee Westmaas, Jamie Ostroff

**Affiliations:** 1 Fred Hutchinson Cancer Research Center Division of Public Health Sciences Seattle, WA United States; 2 Department of Global Health University of Washington Seattle, WA United States; 3 American Cancer Society Atlanta, GA United States; 4 Memorial Sloan Kettering New York City, NY United States

**Keywords:** smartphone app, mHealth, tobacco, smoking, cancer patient

## Abstract

**Background:**

Persistent smoking after a cancer diagnosis predicts worse treatment outcomes and mortality, but access to effective smoking cessation interventions is limited. Smartphone apps can address this problem by providing a highly accessible, low-cost smoking cessation intervention designed for patients with a recent cancer diagnosis.

**Objective:**

This study aimed to summarize our development process and report the trial design, feasibility, participant acceptability, preliminary effectiveness, and impact on processes of change (eg, cancer stigma) of the first-known smoking cessation smartphone app targeted for cancer patients.

**Methods:**

We used an agile, user-centered design framework to develop a fully automated smartphone app called Quit2Heal that provided skills training and stories from cancer survivors focusing on coping with internalized shame, cancer stigma, depression, and anxiety as core triggers of smoking. Quit2Heal was compared with the National Cancer Institute’s QuitGuide, a widely used stop smoking app for the general population, in a pilot double-blinded randomized trial with a 2-month follow-up period. Participants were 59 adult smokers diagnosed with cancer within the past 12 months and recruited through 2 cancer center care networks and social media over a 12-month period. The most common types of cancer diagnosed were lung (21/59, 36%) and breast (10/59, 17%) cancers. The 2-month follow-up survey retention rate was 92% (54/59) and did not differ by study arm (*P*=.15).

**Results:**

Compared with QuitGuide participants, Quit2Heal participants were more satisfied with their assigned app (90% [19/21] for Quit2Heal vs 65% [17/26] for QuitGuide; *P*=.047) and were more likely to report that the app assigned to them was made for someone like them (86% [18/21] for Quit2Heal vs 62% [16/26] for QuitGuide; *P*=.04). Quit2Heal participants opened their app a greater number of times during the 2-month trial period, although this difference was not statistically significant (mean 10.0, SD 14.40 for Quit2Heal vs mean 6.1, SD 5.3 for QuitGuide; *P*=.33). Self-reported 30-day point prevalence quit rates at the 2-month follow-up were 20% (5/25) for Quit2Heal versus 7% (2/29) for QuitGuide (odds ratio 5.16, 95% CI 0.71-37.29; *P*=.10). Quit2Heal participants also showed greater improvement in internalized shame, cancer stigma, depression, and anxiety, although these were not statistically significant (all *P*>.05).

**Conclusions:**

In a pilot randomized trial with a high short-term retention rate, Quit2Heal showed promising acceptability and effectiveness for helping cancer patients stop smoking. Testing in a full-scale randomized controlled trial with a longer follow-up period and a larger sample size is required to test the effectiveness, mediators, and moderators of this promising digital cessation intervention.

**Trial Registration:**

ClinicalTrials.gov NCT03600038; https://clinicaltrials.gov/ct2/show/NCT03600038

## Introduction

In the United States, 15% to 54% of cancer patients are cigarette smokers at the time of their diagnosis [1-4]. Compared with patients who quit smoking after their diagnosis, cancer patients who remain smokers have worse treatment outcomes, including 2 to 4 times higher risk of nonresponse to radiation [5-7], decreased efficacy and tolerance of chemotherapy [8,9], and 2 to 3.5 times higher risk of postoperative complications such as necrosis [10]. Regardless of the type of cancer diagnoses, patients who continue to smoke after diagnosis have 1.5 to 4 times higher risk of a second primary oral, oropharyngeal, esophageal, stomach, lung, or hematological cancer [11,12]. Finally, the mortality rate among cancer patients who continue smoking is 1.3 to 2.4 times higher across all types of cancer [5,13,14]. In contrast, quitting smoking after receiving a cancer diagnosis greatly reduces the risk of poor treatment outcomes [5,15] and of a second primary cancer [11,12] and lowers the mortality rates [5,13,14]. This broad body of evidence has contributed to the Surgeon General’s conclusion that quitting smoking after a diagnosis will vastly improve the prognosis of patients with cancer [16] and to the National Comprehensive Cancer Network’s (NCCN’s) recommendation that every cancer patient who smokes must be offered evidence-based cessation intervention [17].

Unfortunately, up to 80% of smokers with cancer continue to smoke after their diagnosis [2,18-20]. Moreover, 15% to 25% of those who do quit after a cancer diagnosis will return to smoking within 12 months [4,21,22]. Despite the NCCN recommendation, tobacco treatment delivery for cancer patients in the Unites States is inadequate because of several barriers that limit patients’ access to cessation treatment. For example, only 39% of oncologists routinely provide tobacco cessation treatment to patients or at least refer them to a tobacco treatment program [23]. Moreover, only 20% of the National Cancer Institute (NCI)–designated cancer centers have a tobacco treatment provider [24]. Barriers to access also include the lack of insurance coverage; the lack of clinical staff training and time; and the lack of systems for universal assessment, referral, and integration of cessation service into routine cancer care [23,24].

In direct response to this need, the US NCI created a Moonshot-funded Cancer Center Cessation Initiative (C3I) to support the implementation of evidence-based cessation interventions at 42 NCI-designated cancer centers [25]. The C3I is making progress in implementing treatment programs within the 42 participating NCI cancer centers [26] but is hindered by the complex tobacco treatment delivery challenges of limited hospital resources, inadequate clinical staff training, and clinic workflows [26]. The much larger challenge is the fact that a mere 15% of cancer patients receive their cancer treatment at NCI-designated cancer centers [27]. Overall, these challenges demonstrate the need for broader methods to reach cancer patients who smoke.

One method for all smokers with cancer to access effective and low-cost smoking cessation treatment is via smartphone-based smoking cessation software apps [28-30]. Apps do not require provider training, reimbursement for cessation interventions, or integration into complex hospital systems (eg, apps can be freely accessed on an app store), and they are available anytime at arm’s reach [28-30]. Apps have potentially high population-level reach to cancer patients—especially given that over three-quarters (76%) of all smokers own smartphones, and 68% of adults aged 55 to 74 years own smartphones [31,32].

Smartphone apps for smoking cessation are showing solid promise among the general population of smokers [33]. For example, a 4-country trial (N=684), with 85% outcome data retention at the 6-month follow-up, showed that an app combining provision of quitting options with supportive and motivational messages and a quitting benefits tracker was over 2 times more effective than an informational cessation app without these features (10.2% quit rate vs 4.8% quit rate; risk ratio=2.02; 95% CI 1.08-3.81) [34]. Building on the promise of apps for the general population of smokers, a targeted intervention can address the unique processes that impede cessation among cancer patients, including shame about being a smoker, cancer stigma (feeling socially rejected for having caused one’s cancer), depression, and anxiety [35-44]. However, there are no randomized trials evaluating the efficacy of *any* smoking cessation smartphone app among adult cancer patients who smoke.

To address this knowledge gap, we developed a smartphone app, called Quit2Heal*,* that is specifically designed to help cancer patients stop smoking. Quit2Heal was compared with NCI’s QuitGuide, a widely used stop smoking app, in a pilot randomized trial of 59 US adult smokers recently diagnosed with cancer. The objective of this study was to summarize our development process and report the pilot trial recruitment, retention, participant acceptability, preliminary effectiveness, and impact on the hypothesized processes of change (eg, cancer stigma) of the first-known smoking cessation smartphone app targeted for cancer patients.

## Methods

### Development of Quit2Heal—A Smartphone App Designed to Help Cancer Patients Stop Smoking

We used an iterative, user-centered design approach [45] to develop an app designed to help cancer patients quit smoking. Our starting point for the development work was a smartphone app called iCanQuit, which we are currently testing in a large randomized trial for smoking cessation in a general population of adult smokers (NCT02724462). iCanQuit teaches skills for coping with smoking urges, staying motivated, and preventing relapse. To guide the adaptation of iCanQuit for cancer patients who smoke, we interviewed smoking cessation clinicians at 4 NCI-designated cancer centers across the United States and reviewed (1) the empirical studies on the factors that influence quitting smoking in cancer patients (ie, shame, stigma, depression, and anxiety [35-37]), (2) the NCCN Clinical Practice Guidelines in Oncology for smoking cessation [17], (3) the intervention content from published protocols and trials for smoking cessation of cancer patients [41,46-49], and (4) clinical intervention protocols of smoking cessation of cancer patients. We then conducted in-depth, in-person interviews of 6 smokers currently in treatment for cancer (2 attributable and 4 not attributable to smoking) and 3 caregivers of current cancer patients who smoke. The major themes derived from these interviews were lack of knowledge regarding the effects of smoking on cancer-related outcomes, mental health problems (ie, depression and anxiety) associated with smoking and cancer, shame about smoking, feeling stigmatized about being a cancer patient who smoked, and fears of seeking support from and/or discomfort discussing smoking and quitting with cancer treatment providers.

Our formative research led us to iteratively develop content on the (1) consequences of continued smoking versus quitting smoking for health domains such as daily functioning and cancer treatment outcomes, (2) skills for coping with depression and anxiety often associated with a cancer diagnosis, (3) self-compassion exercises for coping with cancer-related stigma and internalized shame, (4) advice on how to seek support for quitting smoking from cancer treatment providers (eg, oncologist), and (5) testimonials from cancer survivors describing how quitting smoking has allowed them to live more meaningful lives. The wireframes created by our user experience designer were iterated upon by our team. The content was user tested with 13 smokers currently receiving cancer treatment to get feedback on usability and content in 3 iterative rounds of testing. Our user testing also identified the cancer patients’ choice of the best name for the app, Quit2Heal. After our developer created an alpha version of the Quit2Heal app, the study team identified edits for the content and features as well as any technical bugs.

Our review yielded a beta version that was tested in a 7-day diary study with 5 adult smokers (3 women and 2 men) currently receiving cancer treatment who had varying levels of technical ability and confidence in quitting smoking. The diary study included a 30-min onboarding session, 7 nightly 10-min surveys about each participant’s experience of the app that day, a 10-min call on day 4 to discuss their impressions of the app so far, and a 45-min exit interview about their overall experience and the usability of the app. All participants rated the app as highly useful overall, were very satisfied overall, and would recommend the app to other cancer patients who smoke. They all liked the 5 content areas created specifically for cancer patients who smoke. The major problem area was that they were not clear where to start the app’s program. Our remedies included (1) adding an introduction with screenshots showing how to begin the program and (2) graying out the sections that come later in the program until they become available. After minor usability concerns were remedied, the final version of Quit2Heal was ready for testing in the pilot randomized controlled trial (RCT) described herein.

### Participants, Recruitment, and Enrollment

Participants with the following eligibility criteria were included in the study: (1) aged 18 years or above, (2) diagnosed with cancer within the past 12 months or currently receiving cancer treatment or planning to receive cancer treatment in the next 3 months (consistent with prior trials of smoking cessation in cancer patients [46,50]), (3) smoked a cigarette (even a puff) in the past 30 days, (4) interested in learning skills to quit smoking, (5) willing to be randomly assigned to either smartphone app, (6) living in the United States and planning to remain for the next 2 months, (7) having at least daily access to their own smartphone, (8) knowing how to download a smartphone app, (9) willing and able to read English, (10) not currently using smoking cessation medications or enrolled in another smoking cessation program, and (11) have never used the NCI’s QuitGuide app, (12) willingness to complete 1 survey at the 2-month follow-up, and (13) provision of email address, phone number, and mailing address. (Criterion 12 and 13 were included to increase follow-up retention.)

**Figure 1:**
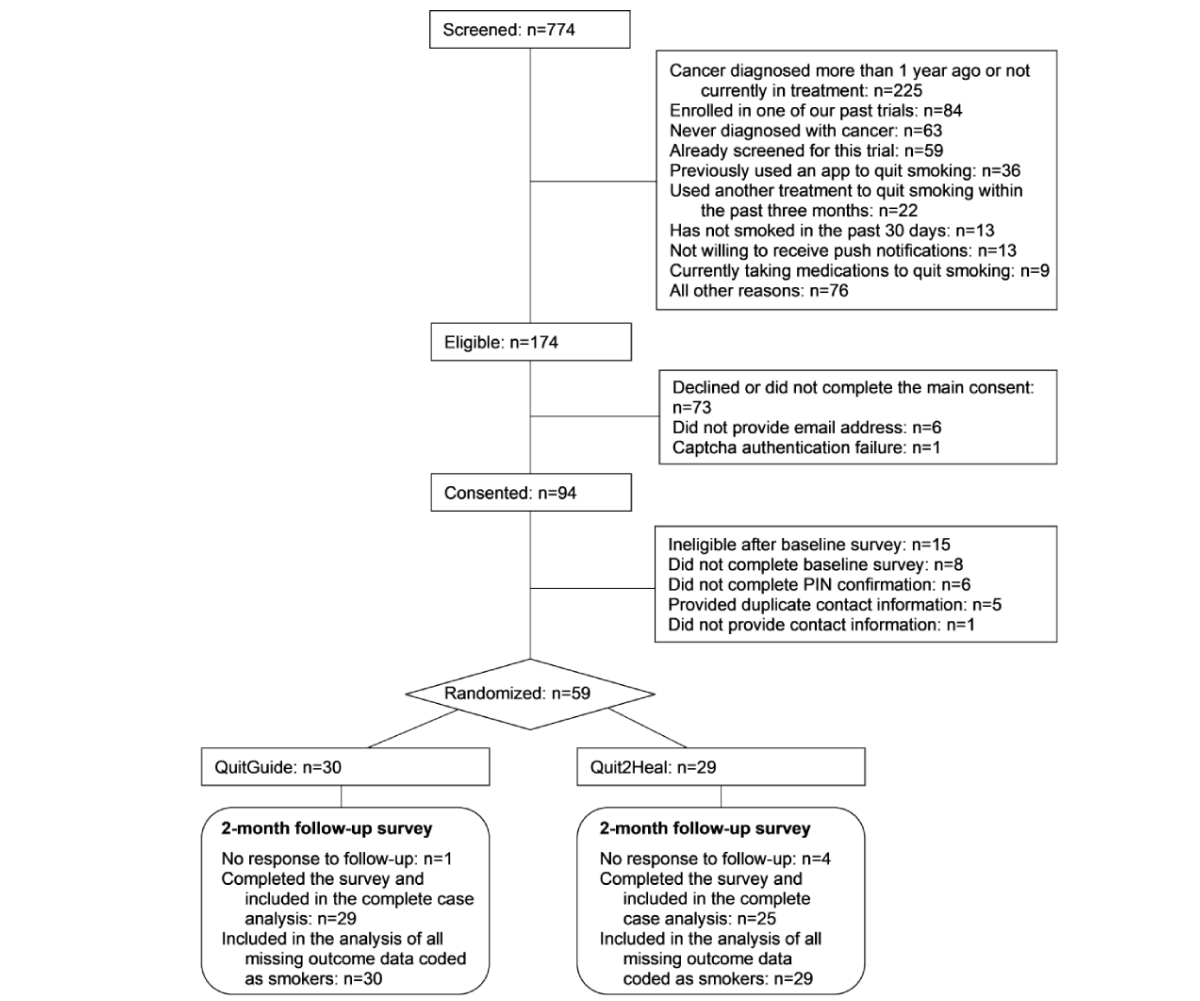
Participant flow diagram.

The study participant flow diagram is shown in Figure 1. Participants were recruited nationally over a 12-month period from April 2, 2018, to April 1, 2019, through social media (primarily Facebook Ads) and 2 US cancer centers (Memorial Sloan Kettering Cancer Center and Seattle Cancer Care Alliance [SCCA]/Fred Hutch Cancer Research Center and their affiliated clinics) via clinic flyers, brochures, waiting room television (TV) screen ticker-tape messages, and emailing the study flyer to current cancer patients who smoke (identified through electronic medical records). Some Facebook Ads were designed for racial and ethnic minorities as well as men. Enrollment was limited to no more than 80% (47/59) non-Hispanic white and 75% (44/59) female participants to ensure the inclusion of racial/ethnic minority and male participants. All interested individuals were directed to the study website to learn more about the study and complete an encrypted Web-based screening survey. Those who were eligible were instantly sent an email inviting them to provide informed consent and complete the encrypted baseline assessment. As the enrollment occurred via the Web, additional actions were taken to ensure that the enrollees were actually eligible for participating in the study. These included CAPTCHA (Completely Automated Public Turing test to tell Computers and Humans Apart) authentication, review of Internet Protocol addresses for duplicates or non-US origin, review of survey logs for suspicious response times (<90 seconds to complete screening or <10 min to complete baseline survey), and review of mailing addresses and phone numbers to check for prior enrollment in one of our previous studies. Those not eligible were not enrolled.

Our original recruitment goal was 200 participants (100 per arm) based on our experience with recruiting 200 participants in prior pilot randomized trials of mobile health (mHealth) and electronic health (eHealth) for smoking cessation for the *general* population of smokers [51,52]. However, by 2 months into this pilot trial’s recruitment period, the Facebook Ad algorithms determined that the cost of Facebook Ads to randomize each cancer patient who smokes was 16 times higher than the cost of Facebook Ads to randomize each smoker from the general population (ie, US $213.25 vs US $13.60). Consequently, to meet our limited pilot budget and complete the recruitment within the funding period, we downward adjusted our recruitment goal to 60. The recruitment sources for the enrolled sample of 59 participants were as follows: 36 participants from Facebook Ads, 3 from all other social media (eg, Craigslist), 1 from a TV news segment, and 19 from cancer care clinics. For reporting readily comparable Facebook recruitment metrics [53,54], the Facebook cost per click, result rate (formerly called *conversion rate*), and impressions were US $0.52, 0.003%, and 714,862, respectively.

A total of 2 reminder emails were sent over a 14-day period to individuals who did not respond to the initial email invitation. Individuals who did not consent or complete the enrollment process within the 14-day period were sent an email indicating that they were not enrolled. Participants not enrolled (or ineligible) were referred to *Smokefree.gov* and *800-QUIT-NOW*. Participants randomized to the trial were emailed a secured link to download their randomly assigned app (either Quit2Heal or QuitGuide) on either an Android smartphone or an iPhone. All study activities were reviewed and approved by the institutional review boards of the Fred Hutchinson Cancer Research Center and Memorial Sloan Kettering Cancer Center. The trial was registered on ClinicalTrials.gov (registration number NCT03600038).

### Randomization Procedures

The enrolled participants were randomized (1:1) to either the experimental intervention (Quit2Heal, n=29) or the control intervention (QuitGuide, n=30). We used computer-generated randomly permuted block randomization, stratified by Heaviness of Smoking Index (score>4 [55]), confidence in being smoke-free >70 (on a scale of 0-100), and recruitment method (ie, clinic vs social media). The randomization assignment was concealed from participants throughout the entire trial. Neither the research staff nor the study participants had access to the upcoming randomized study arm assignments. The study staff and investigators were blind to the random assignment throughout the trial.

To ensure participants were blinded to their assigned intervention, each app was branded as Quit2Heal. Contamination between the interventions was avoided with a unique username and password provided only to the study participant and by having an eligibility criterion of not having family, friends, or other household members participating.

### Experimental Intervention

Quit2Heal [56,57] is specifically designed to help cancer patients stop smoking by providing skills to cope with cancer-related shame, stigma, depression, anxiety, and cancer-specific health consequences of continued smoking versus quitting. After setting up a personalized quit plan where users can learn about the Food and Drug Administration (FDA)–approved cessation medications they can obtain on their own, users are taken to the home screen where they can progress through all 9 levels of the intervention content, receive on-demand help in coping with smoking urges, track the number of cigarettes smoked daily, and track how many urges they let pass without smoking. The program is self-paced, and the content is unlocked in a sequential manner. For the first 5 levels, exercises are unlocked immediately after the prior exercise is complete. For the last 4 levels, the next level will not unlock until users record 7 consecutive smoke-free days. If a participant lapses (eg, records having smoked a cigarette), the program encourages (but will not require) the participant to set a new quit date and return to the first 5 levels for preparation.

The first 5 levels contain content and exercises designed to prepare the users for their chosen quit day. Level 1, *Becoming an Urge Expert*, introduces the main features of the app and introduces a fictional tobacco treatment *guide* who specializes in helping cancer patients quit smoking. The guide navigates the user through the app and teaches skills for coping with cancer-related depression, anxiety, shame, stigma, and common triggers to smoke (eg, being around other smokers). An example of a skill for coping with cancer-related depression is having the user track small things that the user was grateful for on that day (eg, less pain and being able to attend a child’s birthday party). Levels 2 to 4 contain 26 exercises teaching skills to cope with cravings, emotions, and thoughts that trigger smoking. Level 5, *Becoming a Kindness Expert* contains 9 exercises designed to help the users develop self-compassion for themselves for shame and stigma about being a cancer patient who smokes. An example of the skill for coping with shame is an exercise to forgive the user’s younger self for choosing to start smoking. An example of the skill for coping with stigma is an exercise in shifting perspective, having kind words for the people who the user perceives had stigmatized them.

The last 4 levels contain content and exercises designed to help the user stay smoke-free after their quit date. These levels contain 25 exercises that focus on coping with cancer-related depression and anxiety, withdrawal symptoms, slips, and potential weight gain and building smoke-free life activities. All levels contain at least one *user story* (testimonial) presented by fictitious cancer patients who quit smoking; how they overcame challenges, including cancer-related shame and stigma; and how quitting has helped them live more meaningful lives (eg, spending more time with family).

Through the main menu, participants access the education section, which has 3 components. The first component educates on the negative consequences of continuing to smoke after a cancer diagnosis: (1) impacts on radiation, chemotherapy, and postsurgical recovery; (2) risk of second primary cancers; and (3) mortality. The second component educates on the positive consequences of quitting smoking after a cancer diagnosis: (1) improved treatment outcomes, (2) lesser chance of a second primary cancer, and (3) lower chance of mortality. The third section contains education on how to talk to a cancer care provider about the user’s smoking and ask for the provider’s assistance and support in quitting—which can help reduce shame and stigma. The participants can edit their quit plan, review their progress (eg, smoke-free days), and view the badges they earned for making progress in the program.

### Comparison Intervention

The comparison was NCI’s QuitGuide app [58,59] which, with the NCI’s permission, we posted on the Google Play and Apple Store in a blinded format branded as Quit2Heal. We selected QuitGuide as the comparison because (1) QuitGuide is a smartphone app—the treatment delivery modality identical to our experimental Quit2Heal app and, thus, avoids confounding treatment *content* with treatment delivery *modality*; (2) QuitGuide’s content is based directly on the NCI’s Smokefree.gov website, a well-established eHealth intervention resource recommended by the NCCN Clinical Practice Guidelines for cancer patients’ smoking cessation [17]; (3) QuitGuide is one of the few apps (of over 500 available) that follow the US Clinical Practice Guidelines [60]; and (4) QuitGuide is nonproprietary and free to the public, providing maximal transparency, accessibility, and replicability.

QuitGuide is a *non-targeted* smoking cessation app designed for the *general population of smokers*, with 4 sections of content: (1) *Thinking about quitting*, which focuses on motivations to quit by encouraging the users to list reasons for quitting and providing information on the general health consequences of smoking and quitting; (2) *Preparing to Quit*, which helps users develop a customized quit plan; identify smoking behavior, triggers, and reasons for being smoke-free; and identify social support for quitting and provides information on FDA-approved medications to quit smoking; (3) *Quitting*, which teaches skills for avoiding cravings to smoke, such as finding replacement behaviors (eg, chewing on carrot sticks) and staying busy; and (4) *Staying Quit*, which presents tips, motivations, and actions to stay smoke-free and skills for coping with slips via fighting cravings and trying to be positive.

Both interventions were available for log in at any time after randomization. Neither was modified during the study (the apps used in this study are available for download, with tester usernames and passwords available upon request [56-59]).

### Follow-Up Data Collection

The procedures for follow-up data collection were modeled after the procedures that have been successful in our previous trials at maximizing data retention [52,61,62]. Specifically, at 2 months post randomization, participants received US $25 for completing the follow-up survey and an additional US $10 bonus if the encrypted online survey was completed within 24 hours of the initial email invitation to complete the survey. Participants who did not complete the survey online within 12 days were sequentially offered opportunities to do so by phone, mailed survey, and finally, for main outcomes only, by postcard.

### Measures

#### Baseline Measures

At baseline, participants reported their demographics, cancer (eg, type and stage), alcohol use (Quick Drinking Screen [63]), current and past tobacco use, nicotine dependence (Fagerstrom Test for Nicotine Dependence; FTND [64]), confidence in quitting, and smoking in the social environment.

#### Treatment Utilization

Utilization was assessed via data logged automatically by the secured server on how many times the app was opened during the 60 days after randomization. Owing to a database error, these data were only available from the 32 participants who were randomized after June 29, 2018.

#### Treatment Satisfaction

Treatment satisfaction outcomes were the extent to which a participant (1) was overall satisfied with the assigned app, (2) would recommend the assigned app to a friend, and (3) believed that the assigned app was made for someone like them. The response choices for all items ranged from *not at all* (1) to *very much* (5) and were dichotomized such that a threshold of *somewhat* (3) or higher represented satisfaction.

#### Process Measures

The brief process measures, assessed at baseline and the 2-month follow-up, were internalized shame (5-item internalized shame subscale of the Social Impact Scale [65]), internalized stigma (9-item internalized stigma subscale of the Lung Cancer Stigma Inventory [35]), depression (10-item Center for Epidemiologic Studies Depression Scale; CES-D [66]), and generalized anxiety (7-item Generalized Anxiety Disorder; GAD-7 [67]). Internalized shame refers to the perception that one’s illness sets one apart from others who are well and feeling a need for secrecy about the illness [65]. A sample scale item is “I feel a need to keep my illness secret.” Internalized stigma refers to the internalized experience of rejection, blame, and devaluation based on the assumption that one has caused one’s illness [35]. We modified the internalized stigma subscale of the Lung Cancer Stigma Inventory so that it focused on cancer broadly (rather than only lung cancer). A sample scale item is “I blame myself for having cancer.”

#### Smoking Cessation

For scientific rigor and comparability with other low-intensity behavioral intervention trials [68,69], the cessation outcome was self-reported 30-day point prevalence abstinence (ie, no smoking at all in the past 30 days), which was calculated based on the response to the question “When was the last time you smoked, or even tried, a cigarette?” Owing to the cost and low demand characteristics for false reporting, the Society for Research on Nicotine and Tobacco Subcommittee on Biochemical Verification recommends that biochemical confirmation is unnecessary in population-based studies with limited face-to-face contact and in studies where the optimal data collection methods are remote (eg, telephone) [70]. Self-reported smoking is a standard method for assessing the efficacy of low-intensity interventions [68,69].

### Statistical Analysis

The demographic characteristics, smoking behavior, and process measures at baseline were compared between the study groups using 2-sample *t* tests for continuous variables and Fisher exact test for binary variables. All participants were analyzed using intent to treat in the study arm to which they were randomized. The abstinence outcomes were calculated as both complete case and missing equals smoking imputation.

We used logistic regression models to analyze the differences between the treatment arms on binary cessation and satisfaction outcomes. A negative binomial model was used to analyze the right-skewed app utilization data. Linear models were used to analyze the changes in process indicator measures, adjusting for baseline value of the measure. All models were adjusted for the 3 variables used in stratified randomization. The models were also adjusted for any baseline characteristic that was both imbalanced between the study arms at baseline (ie, *P*<.10) and associated with the outcome of interest. Statistical tests were 2-sided, with alpha=.05. Analyses were completed using R version 3.6.1 [71] and R library MASS [72].

## Results

### Baseline Characteristics, Balance, and Follow-Up Retention

As shown in Table 1, the overall sample was aged 45.2 years on average and comprised 25% (15/59) male, 78% (46/59) white, 29% (17/59) with high school or less education, and 22% (13/59) who identified as lesbian, gay, or bisexual (LGB). The rates of positive screen for depression (CES-D≥10) and anxiety (GAD-7≥10) were 73% and 39%, respectively. The most common primary cancer diagnoses were lung (21/59, 36%) and breast (10/59, 17%) cancers. The most common stages of cancer were stage I (17/47, 36%) and stage II (14/47, 30%). With regard to smoking characteristics, 51% (30/59) of the sample had high nicotine dependence (ie, FTND score ≥6 [64]) and 56% (33/59) smoked more than half a pack of cigarettes per day. About one-third (19/59, 32%) lived with a partner who smoked. The 2-month follow-up survey retention rate was 92% (54/59) and did not differ by study arm (*P*=.15). Although none of the measured baseline characteristics significantly differed between the study arms (all *P*>.05), the level of education completed trended toward an imbalance (*P*=.07) and was predictive of the cessation outcome; therefore, we adjusted for this variable in subsequent analyses.

**Table 1:** Baseline characteristics of Quit2Heal study participants.

			Total (N=59)^a^	QuitGuide (N=30)^a^	Quit2Heal (N=29)^a^	*P* value
Age (years), mean (SD)			45.2 (12.9)	47.3 (13.5)	42.9 (12.0)	.19
Male, % (n/N)			25 (15/59)	30 (9/30)	21 (6/29)	.60
**Race, % (n/N)**						**.29^b^**
	White		78 (46/59)	77 (23/30)	79 (23/29)	
	Black or African American		12 (7/59)	10 (3/30)	14 (4/29)	
	Native American		2 (1/59)	3 (1/30)	0 (0/29)	
	Asian		2 (1/59)	3 (1/30)	0 (0/29)	
	More than 1 race		3 (2/59)	7 (2/30)	0 (0/29)	
	Unknown race		3 (2/59)	0 (0/30)	7 (2/29)	
	Hispanic		7 (4/59)	3 (1/30)	10 (3/29)	.58
Married, % (n/N)			44 (26/59)	40 (12/30)	48 (14/29)	.71
Working, % (n/N)			46 (27/59)	40 (12/30)	52 (15/29)	.52
High school or less education, % (n/N)			29 (17/59)	17 (5/30)	41 (12/29)	.07
Lesbian, Gay, or Bisexual % (n/N)			22 (13/59)	20 (6/30)	7 (24)	.95
**Mental health**						
	Positive depression screen, % (n/N)		73 (43/59)	70 (21/30)	76 (22/29)	.83
	Positive anxiety screen, % (n/N)		39 (23/59)	30 (9/30)	48 (14/29)	.24
	Internalized shame^c^, mean (SD)		11.3 (4.2)	11.5 (3.8)	11.2 (4.7)	.77
	Cancer-related stigma^d^, mean (SD)		30.1 (11.9)	31.2 (10.4)	28.9 (13.4)	.47
**Cancer-related background**						
	**Cancer diagnosis, % (n/N)**					**.40^e^**
		Lung	36 (21/59)	30 (9/30)	41 (12/29)	
		Breast	17 (10/59)	20 (6/30)	14 (4/29)	
		Skin	7 (4/59)	10 (3/30)	3 (1/29)	
		Cervical	5 (3/59)	3 (1/30)	7 (2/29)	
		Colorectal	3 (2/59)	3 (1/30)	3 (1/29)	
		Leukemia	3 (2/59)	0 (0/30)	7 (2/29)	
		Non-Hodgkin lymphoma	3 (2/59)	0 (0/30)	7 (2/29)	
		Pancreatic	3 (2/59)	7 (2/30)	0 (0/29)	
		Esophageal	2 (1/59)	3 (1/30)	0 (0/29)	
		Liver	2 (1/59)	3 (1/30)	0 (0/29)	
		Prostate	2 (1/59)	0 (0/30)	3 (1/29)	
		Stomach	2 (1/59)	0 (0/30)	3 (1/29)	
		Throat	2 (1/59)	3 (1/30)	0 (0/29)	
		All others	14 (8/59)	17 (5/30)	10 (3/29)	
	**Stage of cancer, % (n/N)**					**.41^f^**
		0	11 (5/47)	5 (1/21)	15 (4/26)	
		I	36 (17/47)	48 (10/21)	27 (7/26)	
		II	30 (14/47)	19 (4/21)	38 (10/26)	
		III	11 (5/47)	5 (1/21)	15 (4/26)	
		IV	13 (6/47)	24 (5/21)	4 (1/26)	
	Months since initial diagnosis, mean (SD)		4.7 (3.5)	4.2 (3.7)	5.3 (3.3)	.23
	**Type of standard cancer treatment completed, % (n/N)**					
		Chemotherapy	51 (21/41)	48 (10/21)	55 (11/20)	.87
		Radiation	41 (17/41)	38 (8/21)	45 (9/20)	.90
		Surgery	44 (18/41)	38 (8/21)	50 (10/20)	.65
		Hormone therapy	12 (5/41)	14 (3/21)	10 (2/20)	>.99
		Stem cell transplant	0 (0/41)	0 (0/21)	0 (0/20)	>.99
		Immunotherapy	0 (0/41)	0 (0/21)	0 (0/20)	>.99
**Smoking behavior**						
	Fagerstrom Test of Nicotine Dependence score, mean (SD)		5.3 (2.2)	5.4 (2.1)	5.1 (2.4)	.69
	High nicotine dependence (FTND ≥6), % (n/N)		51 (30/59)	57 (17/30)	45 (13/29)	.52
	Smokes more than half a pack of cigarettes per day, % (n/N)		56 (33/59)	53 (16/30)	59 (17/29)	.88
	Smoked for 10 or more years, % (n/N)		92 (54/59)	93 (28/30)	90 (26/29)	.97
	Used electronic cigarettes at least once in the past month, % (n/N)		29 (17/59)	37 (11/30)	21 (6/29)	.29
	Made at least one attempt to quit smoking in the past 12 months, % (n/N)		60 (35/58)	69 (20/29)	52 (15/29)	.28
	Number of attempts to quit smoking in the past 12 months, mean (SD)		2.1 (3.3)	2.6 (4.0)	1.6 (2.3)	.25
	Confidence of being smoke-free, mean (SD)		71.7 (25.7)	70.7 (26.5)	72.8 (25.3)	.76
**Friend and partner smoke**						
	Number of close friends who smoke, mean (SD)		2.1 (1.8)	2.3 (1.8)	2.0 (1.9)	.53
	Number of adults at home who smoke, mean (SD)		1.4 (1.1)	1.5 (1.2)	1.3 (1.1)	.52
	Living with partner who smokes, % (n/N)		32 (19/59)	37 (11/30)	28 (8/29)	.64
Heavy alcohol drinker, % (n/N)			5 (3/57)	11 (3/28)	0 (0/29)	.22

^a^Sample size, unless otherwise indicated in the cell.

^b^*P* value from a chi-square test compares distribution of all races between arms. This is an omnibus test, so *P* values for each race are not applicable.

^c^Internalized shame scores range from 5 to 20.

^d^Cancer-related stigma scores range from 9 to 45.

^e^*P* value from a chi-square test compares distribution of all cancer diagnoses between arms. This is an omnibus test, so *P* values for each diagnosis are not applicable.

^f^Numbers shown indicate that not all participants provided the stage of cancer. *P* value from a Wilcoxon rank sum test compares cancer stage between arms.

### Participant Utilization and Satisfaction

Summarizing from Table 2, compared with QuitGuide participants, Quit2Heal participants (1) opened their app a greater number of times during the 2-month trial period, although this difference was not statistically significant (mean 10.0, SD 14.4 vs mean 6.1, SD 5.3; *P*=.33); (2) used the app for more minutes per session, although this was also not statistically significant (mean 3.9, SD 3.2 vs mean 2.7, SD 2.1; *P*=.07); (3) were more satisfied with their assigned app (19/21, 90% vs 17/26, 65%; *P*=.047); and (4) were more likely to report that their assigned app was made for someone like them (18/21, 86% vs 16/26, 62%; *P*=.04).

**Table 2:** Primary and secondary study outcomes.

		QuitGuide (N=30)^a^	Quit2Heal (N=29)^a^	*P* value^b^
**Utilization**				
	Logged in at least once, % (n/N)	97 (29/30)	93 (27/29)	.43
	Number of times the app was opened^c,d^, Mean (SD)	6.1 (5.3)^e^	10.0 (14.4)^f^	.33
	Time spent per log in (in min)	2.7 (2.1)^e^	3.9 (3.2)^e^	.07
	Number of days from the first use to the last use^c^, Mean (SD)	19.8 (22.6)^e^	25.1 (19.8)^f^	.32
**Participant acceptability, % (n/N)**				
	Satisfied overall^g^	65 (17/26)	90 (19/21)	.047
	Will recommend to a friend	57 (16/28)	74 (17/23)	.21
	App was made for someone like to me^g^	62 (16/26)	86 (18/21)	.04
**Smoking outcomes at 2 months, % (n/N)**				
	30-day quit rate, using all available outcome data^d^	7 (2/29)	20 (5/25)	.10
	30-day quit rate, missing outcomes coded as smoking^d^	7 (2/30)	17 (5/29)	.17
**Process indicators^h^, Mean (SD)**				
	Change in internalized shame	0.2 (3.5)^i^	−0.5 (4.7)^j^	.27
	Change in cancer-related stigma	−1.3 (8.8)^i^	−3.0 (9.9)^k^	.48
	Change in depression score	−0.9 (6.5)^l^	−3.5 (5.0)^k^	.38
	Change in anxiety score	0.0 (5.6)^m^	−2.8 (6.8)^j^	.56

^a^Sample size, unless otherwise indicated in the cell.

^b^Two-sided *P* values were calculated from regression models adjusted for 3 factors used in stratified randomization: Heaviness of Smoking Index >4, confidence of being smoke-free >70, and recruitment method (clinical vs nonclinical). Unadjusted 2-sided *P* values were very similar.

^c^App opening data are limited to a subset of participants for whom the objective utilization data were available. Owing to a technical error, automatic recording of the utilization data began 2 months after the beginning of the trial recruitment period.

^d^Regression model was adjusted for high school or less education because of its association with the outcome and slight imbalance between arms.

^e^N=15.

^f^N=17.

^g^Responses were dichotomized as “somewhat,” “mostly,” or “very much” versus “not at all” or “a little.”

^h^Process indicators were calculated as follow-up score minus baseline score.

^i^N=29.

^j^N=25.

^k^N=24.

^l^N=27.

^m^N=28.

#### Smoking Outcomes

The self-reported 30-day point prevalence quit rate for those who completed the 2-month follow-up was 20% (5/25) for Quit2Heal versus 7% (2/29) for QuitGuide (odds ratio [OR] 5.16, 95% CI 0.71-37.29; *P*=.10). Assuming that the 4 participants missing the 2-month outcome data were smoking (ie, missing=smoking), the 30-day adjusted point prevalence quit rate was 17% (5/29) for Quit2Heal versus 7% (2/30) for QuitGuide (OR 3.87, 95% CI 0.57-26.16; *P*=.17).

#### Processes of Change

From baseline to the 2-month follow-up, Quit2Heal participants also reported greater improvement in internalized shame, cancer stigma, depression, and anxiety, although none of these changes were significant (all *P*>.05).

#### Use of Outside Treatment

The use of outside treatments to quit smoking during the 2-month study period did not differ by study arm: nicotine patch (20% for Quit2Heal vs 31% for QuitGuide; *P*=.34), nicotine gum (12% for Quit2Heal vs 17% for QuitGuide; *P*=.59), varenicline (16% for Quit2Heal vs 17% for QuitGuide; *P*=.89), bupropion (4% for Quit2Heal vs 7% for QuitGuide; *P*=.65), and any behavioral program (4% for Quit2Heal vs 0% for QuitGuide; *P*=.94).

## Discussion

### Study Summary

This paper reports the trial recruitment, retention, participant acceptability, preliminary effectiveness, and impact on processes of change of the first-known smoking cessation smartphone app targeted for cancer patients. In general, the results supported all the aims of the pilot study.

### Recruitment, Retention, and Sample Diversity

The trial provided useful information on recruitment sourcing and budgeting. Social media (primarily Facebook Ads) yielded 66% (39/59) of the study sample, which showed that it was an effective method for recruiting cancer patients who smoke for an mHealth intervention trial. Recruitment of this population via Facebook was worth the investment because it was highly useful for recruitment, and an intervention, if proven effective, could be broadly disseminated through Facebook given its high reach to this population.

The outcome data collection protocol yielded a strong overall retention rate of 92%, which is consistent with our past experience with this protocol [62] and affirms its value for future trials of cancer patients who smoke.

The recruitment methods yielded demographics broadly representative of adult cancer patients. There was variability of cancer diagnoses, including cancer diagnoses not typically thought of as caused by smoking but whose treatment would greatly benefit from quitting smoking (eg, breast cancer). In a fully powered trial, it would be worth exploring whether patients with cancers known to be attributable to smoking (eg, lung/head and neck cancers vs patients with all other cancers) are more likely to respond to an app targeted to cancer patients who smoke. The rates of inclusion of participants with mental health problems (eg, depression) and participants who belonged to racial or ethnic minority, were male, identified themselves as LGB, and had high school or less education were encouraging. These sociodemographic groups are typically underrepresented in eHealth and mHealth smoking cessation research [73], although they experience significant disparities in the prevalence and negative health consequences of smoking [74].

### Participant Receptivity and Satisfaction

Although not statistically significant, among those with available log-in data, Quit2Heal participants opened their app more often than QuitGuide participants. Quit2Heal participants were highly satisfied with their app on multiple indicators—substantially more than the QuitGuide participants. Particularly encouraging was the finding that, as an app targeted for cancer patients, 86% of the assigned study participants rated Quit2Heal as being made for someone like them. Taken together, these results suggest that the Quit2Heal content was engaging, acceptable, and seen as relevant by cancer patients.

### Smoking Cessation

Tests of the encouraging quit rates were underpowered as this was a pilot trial. Indeed, the 95% CIs for the ORs for the comparison of quit rates were wide, which is expected in pilot trials with low sample sizes. However, if similar quit rates are found in a fully powered RCT, the overall effect size could have high public health significance.

### Impact on Cancer Patients’ Smoking Processes

The results from Quit2Heal on improvements in internalized shame, cancer stigma, depression, and anxiety are important. They suggest that Quit2Heal may have impacted the processes hypothesized to impede smoking cessation among cancer patients. A future larger trial can determine the extent to which these processes mediate the effects of Quit2Heal on smoking cessation.

### Limitations

The study has several important limitations. As a pilot randomized trial, the sample size was not powered to detect statistically significant differences in quit rates or to conduct formal moderation or mediation analysis of the hypothesized treatment effects. Moreover, substantial smoking relapse naturally occurs after a 2-month follow-up [69,75], especially among cancer patients who smoke, and therefore, a longer-term follow-up (eg, 12 months) is recommended. Owing to a technical error, automatic recording of utilization data did not occur until 2 months after the beginning of the trial recruitment period. Finally, we relied exclusively on the self-reported abstinence in our estimate of 30-day point prevalence abstinence.

### Future Directions

The study results suggest 3 main lines of future research: (1) provide a definitive test of the effectiveness of smoking cessation of smartphone-delivered Quit2Heal compared with QuitGuide—an app that follows US Clinical Practice Guidelines, (2) demonstrate that the smoking cessation outcomes of Quit2Heal are mediated by processes that impede cancer patients’ cessation (eg, internalized shame and cancer stigma), and (3) explore the baseline moderators of treatment effectiveness.

### Conclusions

In a pilot trial with a high short-term follow-up rate, Quit2Heal showed promising acceptability and effectiveness for helping cancer patients stop smoking. Testing in a full-scale RCT is required to definitively determine the effectiveness of Quit2Heal for smoking cessation.

## Appendix

Multimedia Appendix 1 CONSORT-EHEALTH checklist (V 1.6.1).

## Abbreviations

C3I: Cancer Center Cessation Initiative
CAPTCHA: Completely Automated Public Turing test to tell Computers and Humans Apart
CES-D: Center for Epidemiologic Studies
eHealth: electronic health
FDA: Food and Drug Administration
FTND: Fagerstrom Test of Nicotine Dependence
GAD-7: Generalized Anxiety Disorder-7
LGB: lesbian, gay, or bisexual
mHealth: mobile health
NCCN: National Comprehensive Cancer Network
NCI: National Cancer Institute
OR: odds ratio
RCT: randomized controlled trial
SCCA: Seattle Cancer Care Alliance
TV: television
