# Supplementary material for: A Smartphone App Designed to Help Cancer Patients Stop Smoking: Results From a Pilot Randomized Trial on Feasibility, Acceptability, and Effectiveness
Source: JMIR Form Res. 2020 Jan 17;4(1):e16652. doi: 10.2196/16652 (PMC6996729; doi:10.2196/16652)
Supplement: Multimedia Appendix 1 [file formative_v4i1e16652_app1.pdf]

CONSORT-EHEALTH (V 1.6.1) - Submission/Publication Form

The CONSORT-EHEALTH checklist is intended for authors of randomized trials evaluating web-based and Internet-based applications/interventions, including mobile interventions, electronic games (incl multiplayer games), social media, certain telehealth applications, and other interactive and/or networked electronic applications. Some of the items (e.g. all subitems under item 5 - description of the intervention) may also be applicable for other study designs.

The goal of the CONSORT EHEALTH checklist and guideline is to be
a) a guide for reporting for authors of RCTs,
b) to form a basis for appraisal of an ehealth trial (in terms of validity)

CONSORT-EHEALTH items/subitems are MANDATORY reporting items for studies published in the Journal of Medical Internet Research and other journals / scientific societies endorsing the checklist.

Items numbered 1., 2., 3., 4a., 4b etc are original CONSORT or CONSORT-NPT (non-pharmacologic treatment) items.
Items with Roman numerals (i., ii, iii, iv etc.) are CONSORT-EHEALTH extensions/clarifications.

As the CONSORT-EHEALTH checklist is still considered in a formative stage, we would ask that you also RATE ON A SCALE OF 1-5 how important/useful you feel each item is FOR THE PURPOSE OF THE CHECKLIST and reporting guideline (optional).

Mandatory reporting items are marked with a red *.
In the textboxes, either copy & paste the relevant sections from your manuscript into this form - please include any quotes from your manuscript in QUOTATION MARKS,
or answer directly by providing additional information not in the manuscript, or elaborating on why the item was not relevant for this study.

YOUR ANSWERS WILL BE PUBLISHED AS A SUPPLEMENTARY FILE TO YOUR PUBLICATION IN JMIR AND ARE CONSIDERED PART OF YOUR PUBLICATION (IF ACCEPTED).
Please fill in these questions diligently. Information will not be copyedited, so please use proper spelling and grammar, use correct capitalization, and avoid abbreviations.

DO NOT FORGET TO SAVE AS PDF _AND_ CLICK THE SUBMIT BUTTON SO YOUR ANSWERS ARE IN OUR DATABASE !!!

Citation Suggestion (if you append the pdf as Appendix we suggest to cite this paper in the caption):
Eysenbach G, CONSORT-EHEALTH Group
CONSORT-EHEALTH: Improving and Standardizing Evaluation Reports of Web-based and Mobile Health Interventions
J Med Internet Res 2011;13(4):e126
URL: http://www.jmir.org/2011/4/e126/
doi: 10.2196/jmir.1923
PMID: 22209829

* Required

Your name *
First Last

Jonathan Bricker

Primary Affiliation (short), City, Country *
University of Toronto, Toronto, Canada

Fred Hutchinson Center, Seattle, USA

Your e-mail address *

abc@gmail.com

jbricker@fredhutch.org

Title of your manuscript *

Provide the (draft) title of your manuscript.

Smartphone app to help cancer patients stop smoking: Results from a pilot randomized trial on feasibility, acceptability, and effectiveness

Name of your App/Software/Intervention *

If there is a short and a long/alternate name, write the short name first and add the long name in brackets.

Quit2Heal [Quit2Heal smartphone applic

Evaluated Version (if any)

e.g. "V1", "Release 2017-03-01", "Version 2.0.27913"

Version 1.0.2

Language(s) *

What language is the intervention/app in? If multiple languages are available, separate by comma (e.g. "English, French")

English

URL of your Intervention Website or App

e.g. a direct link to the mobile app on app in appstore (itunes, Google Play), or URL of the website. If the intervention is a DVD or hardware, you can also link to an Amazon page.

https://play.google.com/store/apps/det

URL of an image/screenshot (optional)

Your answer

Accessibility *

Can an enduser access the intervention presently?

- ○ access is free and open
- ◉ access only for special usergroups, not open
- ○ access is open to everyone, but requires payment/subscription/in-app purchases
- ○ app/intervention no longer accessible
- ○ Other:

Primary Medical Indication/Disease/Condition *

e.g. "Stress", "Diabetes", or define the target group in brackets after the condition, e.g. "Autism (Parents of children with)", "Alzheimers (Informal Caregivers of)"

Cancer (Patients who smoke cigarettes)

Primary Outcomes measured in trial *

comma-separated list of primary outcomes reported in the trial

Self-reported 30-day point prevalence ab

Secondary/other outcomes

Are there any other outcomes the intervention is expected to affect?

Participant acceptability/satisfaction with their assigned app

Recommended "Dose" *

What do the instructions for users say on how often the app should be used?

- (x) Approximately Daily
- ( ) Approximately Weekly
- ( ) Approximately Monthly
- ( ) Approximately Yearly
- ( ) "as needed"
- ( ) Other:

Approx. Percentage of Users (starters) still using the app as recommended after 3 months *

- (x) unknown / not evaluated
- ( ) 0-10%
- ( ) 11-20%
- ( ) 21-30%
- ( ) 31-40%
- ( ) 41-50%
- ( ) 51-60%
- ( ) 61-70%
- ( ) 71%-80%
- ( ) 81-90%
- ( ) 91-100%
- ( ) Other:

Overall, was the app/intervention effective? *

- ( ) yes: all primary outcomes were significantly better in intervention group vs control
- (x) partly: SOME primary outcomes were significantly better in intervention group vs control
- ( ) no statistically significant difference between control and intervention
- ( ) potentially harmful: control was significantly better than intervention in one or more outcomes
- ( ) inconclusive: more research is needed
- ( ) Other:

Article Preparation Status/Stage *

At which stage in your article preparation are you currently (at the time you fill in this form)

- ( ) not submitted yet - in early draft status
- ( ) not submitted yet - in late draft status, just before submission
- ( ) submitted to a journal but not reviewed yet
- (•) submitted to a journal and after receiving initial reviewer comments
- ( ) submitted to a journal and accepted, but not published yet
- ( ) published
- ( ) Other:

Journal *

If you already know where you will submit this paper (or if it is already submitted), please provide the journal name (if it is not JMIR, provide the journal name under "other")

- ( ) not submitted yet / unclear where I will submit this
- (•) Journal of Medical Internet Research (JMIR)
- ( ) JMIR mHealth and UHealth
- ( ) JMIR Serious Games
- ( ) JMIR Mental Health
- ( ) JMIR Public Health
- ( ) JMIR Formative Research
- ( ) Other JMIR sister journal
- ( ) Other:

Is this a full powered effectiveness trial or a pilot/feasibility trial? *

- (•) Pilot/feasibility
- ( ) Fully powered

Manuscript tracking number *

If this is a JMIR submission, please provide the manuscript tracking number under "other" (The ms tracking number can be found in the submission acknowledgement email, or when you login as author in JMIR. If the paper is already published in JMIR, then the ms tracking number is the four-digit number at the end of the DOI, to be found at the bottom of each published article in JMIR)

- ( ) no ms number (yet) / not (yet) submitted to / published in JMIR
- (•) Other: ms#16652

TITLE AND ABSTRACT

1a) TITLE: Identification as a randomized trial in the title

1a) Does your paper address CONSORT item 1a? *

I.e does the title contain the phrase "Randomized Controlled Trial"? (if not, explain the reason under "other")

- (x) yes
- ( ) Other:

1a-i) Identify the mode of delivery in the title

Identify the mode of delivery. Preferably use "web-based" and/or "mobile" and/or "electronic game" in the title. Avoid ambiguous terms like "online", "virtual", "interactive". Use "Internet-based" only if Intervention includes non-web-based Internet components (e.g. email), use "computer-based" or "electronic" only if offline products are used. Use "virtual" only in the context of "virtual reality" (3-D worlds). Use "online" only in the context of "online support groups". Complement or substitute product names with broader terms for the class of products (such as "mobile" or "smart phone" instead of "iphone"), especially if the application runs on different platforms.

	1	2	3	4	5	
subitem not at all important	( )	( )	( )	( )	(x)	essential

Does your paper address subitem 1a-i? *

Copy and paste relevant sections from manuscript title (include quotes in quotation marks "like this" to indicate direct quotes from your manuscript), or elaborate on this item by providing additional information not in the ms, or briefly explain why the item is not applicable/relevant for your study

The title includes "Smartphone app"

1a-ii) Non-web-based components or important co-interventions in title

Mention non-web-based components or important co-interventions in title, if any (e.g., "with telephone support").

	1	2	3	4	5	
subitem not at all important	(x)	( )	( )	( )	( )	essential

Does your paper address subitem 1a-ii?

Copy and paste relevant sections from manuscript title (include quotes in quotation marks "like this" to indicate direct quotes from your manuscript), or elaborate on this item by providing additional information not in the ms, or briefly explain why the item is not applicable/relevant for your study

There were no non-web-based components or important co-interventions, so this item is not applicable/relevant.

1a-iii) Primary condition or target group in the title

Mention primary condition or target group in the title, if any (e.g., "for children with Type I Diabetes") Example: A Web-based and Mobile Intervention with Telephone Support for Children with Type I Diabetes: Randomized Controlled Trial

	1	2	3	4	5	
subitem not at all important	( )	( )	( )	( )	(x)	essential

Does your paper address subitem 1a-iii? *

Copy and paste relevant sections from manuscript title (include quotes in quotation marks "like this" to indicate direct quotes from your manuscript), or elaborate on this item by providing additional information not in the ms, or briefly explain why the item is not applicable/relevant for your study

The title includes "to help cancer patients stop smoking", identifying the primary target group as cancer patients who smoke

1b) ABSTRACT: Structured summary of trial design, methods, results, and conclusions

NPT extension: Description of experimental treatment, comparator, care providers, centers, and blinding status.

1b-i) Key features/functionalities/components of the intervention and comparator in the METHODS section of the ABSTRACT

Mention key features/functionalities/components of the intervention and comparator in the abstract. If possible, also mention theories and principles used for designing the site. Keep in mind the needs of systematic reviewers and indexers by including important synonyms. (Note: Only report in the abstract what the main paper is reporting. If this information is missing from the main body of text, consider adding it)

	1	2	3	4	5	
subitem not at all important	○	○	○	◉	○	essential

Does your paper address subitem 1b-i? *

Copy and paste relevant sections from the manuscript abstract (include quotes in quotation marks "like this" to indicate direct quotes from your manuscript), or elaborate on this item by providing additional information not in the ms, or briefly explain why the item is not applicable/relevant for your study

"We used an agile, user-centered design framework to develop a smartphone app called "Quit2Heal", specifically designed to help cancer patients stop smoking by providing skills training and stories from cancer survivors that focus on coping with internalized shame, cancer stigma, depression, and anxiety as core triggers of smoking. Quit2Heal was compared with NCI's QuitGuide, a widely used stop smoking app for the general population, in a pilot double-blinded randomized trial with a 2-month follow-up period."

1b-ii) Level of human involvement in the METHODS section of the ABSTRACT

Clarify the level of human involvement in the abstract, e.g., use phrases like "fully automated" vs. "therapist/nurse/care provider/physician-assisted" (mention number and expertise of providers involved, if any). (Note: Only report in the abstract what the main paper is reporting. If this information is missing from the main body of text, consider adding it)

	1	2	3	4	5	
subitem not at all important	○	◉	○	○	○	essential

Does your paper address subitem 1b-ii?

Copy and paste relevant sections from the manuscript abstract (include quotes in quotation marks "like this" to indicate direct quotes from your manuscript), or elaborate on this item by providing additional information not in the ms, or briefly explain why the item is not applicable/relevant for your study

There was no human involvement; "fully automated."

1b-iii) Open vs. closed, web-based (self-assessment) vs. face-to-face assessments in the METHODS section of the ABSTRACT

Mention how participants were recruited (online vs. offline), e.g., from an open access website or from a clinic or a closed online user group (closed usergroup trial), and clarify if this was a purely web-based trial, or there were face-to-face components (as part of the intervention or for assessment). Clearly say if outcomes were self-assessed through questionnaires (as common in web-based trials). Note: In traditional offline trials, an open trial (open-label trial) is a type of clinical trial in which both the researchers and participants know which treatment is being administered. To avoid confusion, use “blinded” or “unblinded” to indicated the level of blinding instead of “open”, as “open” in web-based trials usually refers to “open access” (i.e. participants can self-enrol). (Note: Only report in the abstract what the main paper is reporting. If this information is missing from the main body of text, consider adding it)

	1	2	3	4	5	
subitem not at all important	○	○	○	●	○	essential

Does your paper address subitem 1b-iii?

Copy and paste relevant sections from the manuscript abstract (include quotes in quotation marks "like this" to indicate direct quotes from your manuscript), or elaborate on this item by providing additional information not in the ms, or briefly explain why the item is not applicable/relevant for your study

Yes, we report that it is a self-reported outcome survey.

1b-iv) RESULTS section in abstract must contain use data

Report number of participants enrolled/assessed in each group, the use/uptake of the intervention (e.g., attrition/adherence metrics, use over time, number of logins etc.), in addition to primary/secondary outcomes. (Note: Only report in the abstract what the main paper is reporting. If this information is missing from the main body of text, consider adding it)

	1	2	3	4	5	
subitem not at all important	○	○	○	○	●	essential

Does your paper address subitem 1b-iv?

Copy and paste relevant sections from the manuscript abstract (include quotes in quotation marks "like this" to indicate direct quotes from your manuscript), or elaborate on this item by providing additional information not in the ms, or briefly explain why the item is not applicable/relevant for your study

"Compared to QuitGuide participants, Quit2Heal participants: (1) were more satisfied with their assigned app (90% vs. 65%; P =.047), (2) were more likely to report that their assigned app was made for someone like them (86% vs. 62%; P = .04), and (3) opened their app more times during the 2-month trial period, although this difference was not statistically significant [M = 10.0 (SD = 14.40) vs. 6.1 (SD =5.3); P =.33]. The self-reported 30-day point prevalence quit rate at the 2-month follow-up was 20% for Quit2Heal vs. 7% for QuitGuide (OR=5.16; 95% CI: 0.71, 37.29; p=.104)."

1b-v) CONCLUSIONS/DISCUSSION in abstract for negative trials

Conclusions/Discussions in abstract for negative trials: Discuss the primary outcome - if the trial is negative (primary outcome not changed), and the intervention was not used, discuss whether negative results are attributable to lack of uptake and discuss reasons. (Note: Only report in the abstract what the main paper is reporting. If this information is missing from the main body of text, consider adding it)

	1	2	3	4	5	
subitem not at all important	○	○	○	○	●	essential

Does your paper address subitem 1b-v?

Copy and paste relevant sections from the manuscript abstract (include quotes in quotation marks "like this" to indicate direct quotes from your manuscript), or elaborate on this item by providing additional information not in the ms, or briefly explain why the item is not applicable/relevant for your study

"In a pilot trial with a high short-term retention rate, Quit2Heal had promising acceptability and effectiveness for helping cancer patients stop smoking."

INTRODUCTION

2a) In INTRODUCTION: Scientific background and explanation of rationale

2a-i) Problem and the type of system/solution

Describe the problem and the type of system/solution that is object of the study: intended as stand-alone intervention vs. incorporated in broader health care program? Intended for a particular patient population? Goals of the intervention, e.g., being more cost-effective to other interventions, replace or complement other solutions? (Note: Details about the intervention are provided in “Methods” under 5)

	1	2	3	4	5	
subitem not at all important	○	○	○	◉	○	essential

Does your paper address subitem 2a-i? *

Copy and paste relevant sections from the manuscript (include quotes in quotation marks "like this" to indicate direct quotes from your manuscript), or elaborate on this item by providing additional information not in the ms, or briefly explain why the item is not applicable/relevant for your study

"In the US, 15% to 54% of cancer patients are cigarette smokers at the time of their diagnosis [1-4]. Compared to patients who quit smoking after their diagnosis, cancer patients who remain smokers have worse treatment outcomes including 2 to 4 times higher risk of nonresponse to radiation [5-7], decreased efficacy and tolerance of chemotherapy [8, 9], and 2 to 3.5 times higher risk of postoperative complications such as necrosis [10]. Regardless of the type of cancer an individual is diagnosed with, patients who continue to smoke after diagnosis have 1.5 to 4 times higher risk of second primary oral/pharyngeal, esophageal, stomach, lung, and hematological cancers [11-13]. Finally, the mortality rate among cancer patients who continue smoking is 1.3 to 2.4 times higher across all cancer types [5, 14, 15]. By contrast, quitting smoking after receiving a cancer diagnosis greatly reduces the risk of poor treatment outcomes [5-10, 16] and of a second primary cancer [11-13], and lowers mortality rates [5, 14, 15]." "Building on the promise of apps for the general population of smokers, a tailored intervention can address unique processes that impede cessation among cancer patients including: shame about being a smoker, cancer stigma (feeling socially rejected for having caused one’s cancer), depression, and anxiety [36-43]."

2a-ii) Scientific background, rationale: What is known about the (type of) system

Scientific background, rationale: What is known about the (type of) system that is the object of the study (be sure to discuss the use of similar systems for other conditions/diagnoses, if appropiate), motivation for the study, i.e. what are the reasons for and what is the context for this specific study, from which stakeholder viewpoint is the study performed, potential impact of findings [2]. Briefly justify the choice of the comparator.

	1	2	3	4	5	
subitem not at all important	○	○	○	◉	○	essential

Does your paper address subitem 2a-ii? *

Copy and paste relevant sections from the manuscript (include quotes in quotation marks "like this" to indicate direct quotes from your manuscript), or elaborate on this item by providing additional information not in the ms, or briefly explain why the item is not applicable/relevant for your study

"One method for all smokers with cancer to access effective and low-cost smoking cessation treatment is via smartphone-based smoking cessation software applications ("apps") [29-31]. Apps do not require provider training, reimbursement for cessation interventions, or integration into complex hospital systems (e.g., apps can be freely accessed on an app store), and they are available anytime at arm's reach [24, 25]. Apps have potentially high population level reach to cancer patients—especially given that over three quarters (76%) of all smokers own smartphones, and 68% of adults ages 55 to 74 own smartphones [32, 33]. Smartphone apps for smoking cessation are showing solid promise among the general population of smokers [34]."

2b) In INTRODUCTION: Specific objectives or hypotheses

Does your paper address CONSORT subitem 2b? *

Copy and paste relevant sections from the manuscript (include quotes in quotation marks "like this" to indicate direct quotes from your manuscript), or elaborate on this item by providing additional information not in the ms, or briefly explain why the item is not applicable/relevant for your study

"The objective of this paper is to summarize our development process and report pilot trial recruitment, retention, participant acceptability and preliminary effectiveness and impact on hypothesized processes of change (e.g., cancer stigma) of the first-known smartphone app for smoking cessation tailored to cancer patients."

METHODS

3a) Description of trial design (such as parallel, factorial) including allocation ratio

Does your paper address CONSORT subitem 3a? *

Copy and paste relevant sections from the manuscript (include quotes in quotation marks "like this" to indicate direct quotes from your manuscript), or elaborate on this item by providing additional information not in the ms, or briefly explain why the item is not applicable/relevant for your study

"Enrolled participants were randomized (1:1) to either the experimental intervention (Quit2Heal, n = 29) or the control intervention (QuitGuide, n = 30). We used randomly permuted block randomization, stratified by Heaviness of Smoking Index (score > 4[53]), confidence in being smoke-free >70 (on a 0 to 100 scale), and recruitment method (i.e., clinic vs. social media). "

3b) Important changes to methods after trial commencement (such as eligibility criteria), with reasons

Does your paper address CONSORT subitem 3b? *

Copy and paste relevant sections from the manuscript (include quotes in quotation marks "like this" to indicate direct quotes from your manuscript), or elaborate on this item by providing additional information not in the ms, or briefly explain why the item is not applicable/relevant for your study

"Our original recruitment goal was 200 (100 per arm), based on our experience with recruiting 200 participants in prior pilot randomized trials of mHealth and eHealth of smoking cessation for the general population of smokers [51, 52]. However, by two months into this pilot trial's recruitment period, the Facebook ad algorithms determined that the cost of the Facebook ads to recruit each cancer patient who smokes was 16 times higher than the cost of the Facebook ads to recruit each smoker from the general population (i.e., $213.25 vs. $13.60). Consequently, to meet our limited pilot budget and complete the recruitment within the funding period (12 months), we downward adjusted our recruitment goal to 60."

3b-i) Bug fixes, Downtimes, Content Changes

Bug fixes, Downtimes, Content Changes: ehealth systems are often dynamic systems. A description of changes to methods therefore also includes important changes made on the intervention or comparator during the trial (e.g., major bug fixes or changes in the functionality or content) (5-iii) and other “unexpected events” that may have influenced study design such as staff changes, system failures/downtimes, etc. [2].

	1	2	3	4	5	
subitem not at all important	(●)	( )	( )	( )	( )	essential

Does your paper address subitem 3b-i?

Copy and paste relevant sections from the manuscript (include quotes in quotation marks "like this" to indicate direct quotes from your manuscript), or elaborate on this item by providing additional information not in the ms, or briefly explain why the item is not applicable/relevant for your study

There were no important changes made on the intervention or comparator during the trial or other unexpected events that may have influenced study design, so this subitem is not applicable/relevant.

4a) Eligibility criteria for participants

Does your paper address CONSORT subitem 4a? *

Copy and paste relevant sections from the manuscript (include quotes in quotation marks "like this" to indicate direct quotes from your manuscript), or elaborate on this item by providing additional information not in the ms, or briefly explain why the item is not applicable/relevant for your study

"Eligibility criteria: (1) age 18 or older, (2) diagnosed with cancer within the past 12 months or currently receiving cancer treatment or planning to receive cancer treatment in the next 3 months (consistent with prior trials of cancer patient smoking cessation [46, 47, 50]); (3) smoked a cigarette (even a puff) in the past 30 days; (4) interested in learning skills to quit smoking; (5) willing to be randomly assigned to either smartphone application; (6) live in the United States and plan to remain for the next two months; (7) have at least daily access to their own smartphone; (8) know how to download a smartphone application; (9) be willing and able to read English; (10) not currently using smoking cessation medications or enrolled in another smoking cessation program, and (11) have never used the NCI's QuitGuide app. To increase follow-up retention, eligibility criteria also included: (12) being willing to complete one two-month follow-up survey; and (13) providing email, phone number, and mailing address."

4a-i) Computer / Internet literacy

Computer / Internet literacy is often an implicit “de facto” eligibility criterion - this should be explicitly clarified.

	1	2	3	4	5	
subitem not at all important	○	○	◉	○	○	essential

Does your paper address subitem 4a-i?

Copy and paste relevant sections from the manuscript (include quotes in quotation marks "like this" to indicate direct quotes from your manuscript), or elaborate on this item by providing additional information not in the ms, or briefly explain why the item is not applicable/relevant for your study

Eligibility criteria: "(7) have at least daily access to their own smartphone; (8) know how to download a smartphone application" were our indicators of app literacy.

4a-ii) Open vs. closed, web-based vs. face-to-face assessments:

Open vs. closed, web-based vs. face-to-face assessments: Mention how participants were recruited (online vs. offline), e.g., from an open access website or from a clinic, and clarify if this was a purely web-based trial, or there were face-to-face components (as part of the intervention or for assessment), i.e., to what degree got the study team to know the participant. In online-only trials, clarify if participants were quasi-anonymous and whether having multiple identities was possible or whether technical or logistical measures (e.g., cookies, email confirmation, phone calls) were used to detect/prevent these.

	1	2	3	4	5	
subitem not at all important	○	○	○	◉	○	essential

Does your paper address subitem 4a-ii? *

Copy and paste relevant sections from the manuscript (include quotes in quotation marks "like this" to indicate direct quotes from your manuscript), or elaborate on this item by providing additional information not in the ms, or briefly explain why the item is not applicable/relevant for your study

"Participants were recruited nationally over a 12-month period from April 2, 2018 to April 1, 2019 through social media (primarily Facebook Ads) and two US cancer centers (Memorial Sloan Kettering, Seattle Cancer Care Alliance/Fred Hutch, and their affiliated clinics) via clinic fliers, brochures, waiting room television screen ticker-tape messages, and emailing the study flier to current cancer patients who smoke (identified through electronic medical records)." "All interested individuals were directed to the study website to learn more about the study and complete a web-based screening survey. Those who were eligible were instantly sent an email inviting them to provide informed consent and complete the baseline assessment. Since enrollment occurred online, additional actions were taken to ensure enrollees were actually eligible for study participation. These included CAPTCHA authentication, review of IP addresses for duplicates or non-US origin, and review of survey logs for suspicious response times (< 90 seconds to complete screening or < 10 minutes to complete baseline survey), and review of mailing addresses and phone numbers to check for prior enrollment in one of our previous studies." "Procedures for follow-up data collection were modeled after procedures that have been successful in our previous trials at maximizing data retention [52, 55, 56]. Specifically, at two months post-randomization, participants received $25 for completing the follow-up survey and an additional $10 bonus if the online survey was completed within 24 hours of the initial email invitation to complete the survey. Participants who did not complete the survey online within 12 days were sequentially offered opportunities to do so by phone, mailed survey, and finally, for main outcomes only, by postcard."

4a-iii) Information giving during recruitment

Information given during recruitment. Specify how participants were briefed for recruitment and in the informed consent procedures (e.g., publish the informed consent documentation as appendix, see also item X26), as this information may have an effect on user self-selection, user expectation and may also bias results.

	1	2	3	4	5	
subitem not at all important	○	○	◉	○	○	essential

Does your paper address subitem 4a-iii?

Copy and paste relevant sections from the manuscript (include quotes in quotation marks "like this" to indicate direct quotes from your manuscript), or elaborate on this item by providing additional information not in the ms, or briefly explain why the item is not applicable/relevant for your study

"All interested individuals were directed to the study website to learn more about the study and complete a web-based screening survey. Those who were eligible were instantly sent an email inviting them to provide informed consent and complete the baseline assessment. Two reminder emails were sent over a 14-day period to individuals who did not respond to the initial email invitation. Individuals who did not consent or complete the online enrollment process within the 14-day period were sent an email indicating that they were not enrolled in the study. Participants not enrolled (or eligible) were referred to Smokefree.gov and 800-QUIT-NOW."

4b) Settings and locations where the data were collected

Does your paper address CONSORT subitem 4b? *

Copy and paste relevant sections from the manuscript (include quotes in quotation marks "like this" to indicate direct quotes from your manuscript), or elaborate on this item by providing additional information not in the ms, or briefly explain why the item is not applicable/relevant for your study

All data was collected online, by mail, or by telephone by study staff at the Fred Hutchinson Center: "At two months post-randomization, participants received $25 for completing the follow-up survey and an additional $10 bonus if the online survey was completed within 24 hours of the initial email invitation to complete the survey. Participants who did not complete the survey online within 12 days were sequentially offered opportunities to do so by phone, mailed survey, and finally, for main outcomes only, by postcard."

4b-i) Report if outcomes were (self-)assessed through online questionnaires

Clearly report if outcomes were (self-)assessed through online questionnaires (as common in web-based trials) or otherwise.

	1	2	3	4	5	
subitem not at all important	○	○	○	◉	○	essential

Does your paper address subitem 4b-i? *

Copy and paste relevant sections from the manuscript (include quotes in quotation marks "like this" to indicate direct quotes from your manuscript), or elaborate on this item by providing additional information not in the ms, or briefly explain why the item is not applicable/relevant for your study

Outcomes were self-assessed through online questionnaires, or through telephone or mailed survey or postcard for participants who did not respond online: "Participants who did not complete the survey online within 12 days were sequentially offered opportunities to do so by phone, mailed survey, and finally, for main outcomes only, by postcard."

4b-ii) Report how institutional affiliations are displayed

Report how institutional affiliations are displayed to potential participants [on ehealth media], as affiliations with prestigious hospitals or universities may affect volunteer rates, use, and reactions with regards to an intervention.(Not a required item – describe only if this may bias results)

	1	2	3	4	5	
subitem not at all important	○	○	◉	○	○	essential

Does your paper address subitem 4b-ii?

Copy and paste relevant sections from the manuscript (include quotes in quotation marks "like this" to indicate direct quotes from your manuscript), or elaborate on this item by providing additional information not in the ms, or briefly explain why the item is not applicable/relevant for your study

Institutional affiliation was displayed on the recruitment fliers used at the two US cancer centers and in social media: "Participants were recruited nationally over a 12-month period from April 2, 2018 to April 1, 2019 through social media (primarily Facebook Ads) and two US cancer centers (Memorial Sloan Kettering, Seattle Cancer Care Alliance/Fred Hutch, and their affiliated clinics) via clinic fliers, brochures, waiting room television screen ticker-tape messages, and emailing the study flier to current cancer patients who smoke (identified through electronic medical records)."

5) The interventions for each group with sufficient details to allow replication, including how and when they were actually administered

5-i) Mention names, credential, affiliations of the developers, sponsors, and owners

Mention names, credential, affiliations of the developers, sponsors, and owners [6] (if authors/evaluators are owners or developer of the software, this needs to be declared in a "Conflict of interest" section or mentioned elsewhere in the manuscript).

	1	2	3	4	5	
subitem not at all important	○	◉	○	○	○	essential

Does your paper address subitem 5-i?

Copy and paste relevant sections from the manuscript (include quotes in quotation marks "like this" to indicate direct quotes from your manuscript), or elaborate on this item by providing additional information not in the ms, or briefly explain why the item is not applicable/relevant for your study

The paper mentions the sponsor (CVS Health Foundation) in the "Funding" section, and the developer (Moby, Inc.) in the "Acknowledgements" section. The owner is the Fred Hutchinson Center; the authors do not own the app, so there is no conflict of interest.

5-ii) Describe the history/development process

Describe the history/development process of the application and previous formative evaluations (e.g., focus groups, usability testing), as these will have an impact on adoption/use rates and help with interpreting results.

	1	2	3	4	5	
subitem not at all important	○	○	○	○	◉	essential

Does your paper address subitem 5-ii?

Copy and paste relevant sections from the manuscript (include quotes in quotation marks "like this" to indicate direct quotes from your manuscript), or elaborate on this item by providing additional information not in the ms, or briefly explain why the item is not applicable/relevant for your study

"We used an iterative, user-centered design approach [44] to develop an app tailored to help cancer patients quit smoking. Our starting point for the development work was a smartphone app called iCanQuit, which we are currently testing in a large randomized trial for smoking cessation in a general population of adult smokers (NCT02724462)....To guide the adaptation of iCanQuit for cancer patients who smoke, we interviewed smoking cessation clinicians at four cancer centers across the US and reviewed (1) empirical studies on the psychological factors that influence the likelihood of quitting smoking in cancer patients (i.e., shame, stigma, depression, anxiety [36-38]), (2) the NCCN Clinical Practice Guidelines in Oncology for smoking cessation [18], (3) intervention content from published protocols and trials for cancer patient smoking cessation [43, 46-49], and (4) clinical cancer patient smoking cessation intervention protocols. Subsequently, we conducted in-depth, in-person interviews of six smokers who were currently in treatment for cancer and three caregivers of current cancer patients who smoke." "Overall, our formative research led us to iteratively develop ACT-framed content on: (1) the consequences of continued smoking vs. quitting smoking for valued-health domains such as daily functioning and cancer treatment outcomes, (2) ACT acceptance skills for coping with the depression and anxiety often associated with a cancer diagnosis, (3) ACT self-compassion exercises for coping with cancer-related stigma and internalized shame, (4) advice on how to seek support for quitting smoking from healthcare providers (e.g., oncologist), and (5) composite testimonials from cancer survivors describing how quitting smoking has allowed them to live more meaningful lives. Wireframes created by our user experience designer were iterated upon in team meetings. They were then user tested with 13 smokers currently receiving cancer treatment to get feedback on usability and content in three iterative rounds of testing. Our user testing also identified cancer patients' choice of the best name for the app, "Quit2Heal." After our developer created an initial beta version of the Quit2Heal app, the study team reviewed it for several weeks to identify edits for the new content and features, as well as any technical bugs. Our review yielded a second beta version that was tested in a seven-day diary study with five adult smokers (three women, two men) currently receiving cancer treatment who had varying levels of technical ability and confidence in quitting smoking. The diary study included a 30-minute onboarding session, seven nightly 10-minute surveys with closed and open-ended questions about each participant's experience of the app that day, a 10-minute call on day four to discuss their open-ended impressions of the app so far, and a 45-minute exit interview about their overall experience and the usability of the app. All participants rated the app as highly useful overall, were very satisfied overall, and would recommend the app to other cancer patients who smoke. They all liked the five content areas created specifically for cancer patients who smoke. The major problem area was that they were not clear where to start the app's program. Our remedies included: (1) adding an introduction with screen shots showing how to begin the program and (2) greying out the sections that come later in the program until they become available. There were also a few other minor usability concerns that were easily remedied. Following this final round of changes, Quit2Heal was ready for testing in the pilot randomized trial."

5-iii) Revisions and updating

Revisions and updating. Clearly mention the date and/or version number of the application/intervention (and comparator, if applicable) evaluated, or describe whether the intervention underwent major changes during the evaluation process, or whether the development and/or content was “frozen” during the trial. Describe dynamic components such as news feeds or changing content which may have an impact on the replicability of the intervention (for unexpected events see item 3b).

	1	2	3	4	5	
subitem not at all important	○	○	○	○	◉	essential

Does your paper address subitem 5-iii?

Copy and paste relevant sections from the manuscript (include quotes in quotation marks "like this" to indicate direct quotes from your manuscript), or elaborate on this item by providing additional information not in the ms, or briefly explain why the item is not applicable/relevant for your study

The Quit2Heal app was version 1.0.2. "The comparison was NCI’s QuitGuide app version 1.2." Both apps were unchanged/"frozen" during the trial.

5-iv) Quality assurance methods

Provide information on quality assurance methods to ensure accuracy and quality of information provided [1], if applicable.

	1	2	3	4	5	
subitem not at all important	○	○	○	○	◉	essential

Does your paper address subitem 5-iv?

Copy and paste relevant sections from the manuscript (include quotes in quotation marks "like this" to indicate direct quotes from your manuscript), or elaborate on this item by providing additional information not in the ms, or briefly explain why the item is not applicable/relevant for your study

"Randomization assignment was concealed from participants throughout the entire trial. Neither research staff nor study participants had access to upcoming randomized study arm assignments. Study staff and investigators were blind to random assignment throughout the entire duration of the trial."

5-v) Ensure replicability by publishing the source code, and/or providing screenshots/screen-capture video, and/or providing flowcharts of the algorithms used

Ensure replicability by publishing the source code, and/or providing screenshots/screen-capture video, and/or providing flowcharts of the algorithms used. Replicability (i.e., other researchers should in principle be able to replicate the study) is a hallmark of scientific reporting.

	1	2	3	4	5	
subitem not at all important	○	○	○	○	◉	essential

Does your paper address subitem 5-v?

Copy and paste relevant sections from the manuscript (include quotes in quotation marks "like this" to indicate direct quotes from your manuscript), or elaborate on this item by providing additional information not in the ms, or briefly explain why the item is not applicable/relevant for your study

"The source code is published here: "

5-vi) Digital preservation

Digital preservation: Provide the URL of the application, but as the intervention is likely to change or disappear over the course of the years; also make sure the intervention is archived (Internet Archive, webcitation.org, and/or publishing the source code or screenshots/videos alongside the article). As pages behind login screens cannot be archived, consider creating demo pages which are accessible without login.

	1	2	3	4	5	
subitem not at all important	○	○	○	○	◉	essential

Does your paper address subitem 5-vi?

Copy and paste relevant sections from the manuscript (include quotes in quotation marks "like this" to indicate direct quotes from your manuscript), or elaborate on this item by providing additional information not in the ms, or briefly explain why the item is not applicable/relevant for your study

"Here is the link to download the Quit2Heal app: https://play.google.com/store/apps/details?id=org.fredhutch.quit2healr [Android]; https://apps.apple.com/us/app/id1327059532 [iPhone], and the source code is published here: "
"Here is the link to download the QuitGuide app: https://play.google.com/store/apps/details?id=org.fredhutch.quit2healc [Android], https://apps.apple.com/us/app/quit2heal/id1327061351 [iPhone], and the source code is published here: "

5-vii) Access

Access: Describe how participants accessed the application, in what setting/context, if they had to pay (or were paid) or not, whether they had to be a member of specific group. If known, describe how participants obtained "access to the platform and Internet" [1]. To ensure access for editors/reviewers/readers, consider to provide a "backdoor" login account or demo mode for reviewers/readers to explore the application (also important for archiving purposes, see vi).

	1	2	3	4	5	
subitem not at all important	○	○	○	○	◉	essential

Does your paper address subitem 5-vii? *

Copy and paste relevant sections from the manuscript (include quotes in quotation marks "like this" to indicate direct quotes from your manuscript), or elaborate on this item by providing additional information not in the ms, or briefly explain why the item is not applicable/relevant for your study

"Participants who were randomized into the study were emailed a secured link to download their randomly assigned app (either Quit2Heal or QuitGuide). All participants were emailed identical once weekly reminders to use their assigned intervention. Login access to explore the application provided upon request."

5-viii) Mode of delivery, features/functionalities/components of the intervention and comparator, and the theoretical framework

Describe mode of delivery, features/functionalities/components of the intervention and comparator, and the theoretical framework [6] used to design them (instructional strategy [1], behaviour change techniques, persuasive features, etc., see e.g., [7, 8] for terminology). This includes an in-depth description of the content (including where it is coming from and who developed it) [1]," whether [and how] it is tailored to individual circumstances and allows users to track their progress and receive feedback" [6]. This also includes a description of communication delivery channels and – if computer-mediated communication is a component – whether communication was synchronous or asynchronous [6]. It also includes information on presentation strategies [1], including page design principles, average amount of text on pages, presence of hyperlinks to other resources, etc. [1].

	1	2	3	4	5	
subitem not at all important	○	○	○	○	◉	essential

Does your paper address subitem 5-viii? *

Copy and paste relevant sections from the manuscript (include quotes in quotation marks "like this" to indicate direct quotes from your manuscript), or elaborate on this item by providing additional information not in the ms, or briefly explain why the item is not applicable/relevant for your study

"Quit2Heal (See Multimedia Appendix 1 for screenshots) is a behavioral intervention specifically designed to help cancer patients stop smoking by providing skills training and stories from cancer survivors that focus on coping with internalized shame, cancer stigma, depression, and anxiety as core triggers of smoking. After setting up a personalized quit plan where users have an opportunity to learn about the Food and Drug Administration (FDA)-approved cessation medications that they can obtain on their own, users are taken to the home screen where they are able to progress through all nine levels of the behavioral intervention content, receive on-demand help in coping with smoking urges, track their daily smoking behaviors, and track how many urges they let pass without smoking. The program is self-paced, and content is unlocked in a sequential manner. For the first five levels, exercises are unlocked immediately after the prior exercise is complete. For the last four levels, each level corresponds to seven days of being smoke-free. Therefore, within each level, exercises are unlocked immediately after the prior exercise is viewed; however, the next level will not unlock until users record seven consecutive smoke-free days. If a participant lapses (e.g., recording having smoked a cigarette), the program encourages (but will not require) the participant to set a new quit date and return to first five levels for preparation.

The first five levels contain content and exercises designed to prepare the users for their chosen quit day. Level One, 'Becoming an Urge Expert', introduces the user to the main features of the app, introduces a fictional tobacco treatment specialist who works to help cancer patients quit smoking and guides them through the app, provides skills to help the user identify their smoking triggers, and introduces the idea that distracting oneself from cigarette cravings paradoxically leads to more cravings. Levels Two to Four contain a total of 26 exercises primarily centered on the ACT concept of acceptance and therefore teaches the user skills to allow cravings, emotions, and thoughts that trigger smoking—without acting on them by smoking. Level Five, 'Becoming a Kindness Expert' contains nine exercises designed to help the user develop self-compassion for themselves for being a cancer patient who smokes and for the day-to-day challenges trying to quit smoking or lapsing.

The last four levels contain content and exercises designed to help the user stay smoke-free after their quit date. These levels contain 25 exercises that focus on coping with withdrawal symptoms, slips, depression, anxiety, and potential weight gain, and building a smoke-free life with activities in line with the user's values. All levels contain at least one "user story" presented by a fictitious cancer patient who quit smoking using the skills presented in the app during their cancer treatment, challenges they faced including internalized shame and stigma, and how quitting has helped them do things that deeply matter to them.

On the home screen, participants also have options to anonymously send an email to a quit-smoking coach (emails were answered by our study team), edit their quit plan, review their progress (cigarettes not smoked, money saved), and view the badges they earned throughout the program. Badges were earned for making progress in the program, tracking urges passed, and smoke-free days.

Through the main menu, participants could access information about smoking and cancer, FDA-approved cessation medications, and tips on talking to care providers about smoking and quitting."

5-ix) Describe use parameters

Describe use parameters (e.g., intended “doses” and optimal timing for use). Clarify what instructions or recommendations were given to the user, e.g., regarding timing, frequency, heaviness of use, if any, or was the intervention used ad libitum.

	1	2	3	4	5	
subitem not at all important	○	○	○	◉	○	essential

Does your paper address subitem 5-ix?

Copy and paste relevant sections from the manuscript (include quotes in quotation marks "like this" to indicate direct quotes from your manuscript), or elaborate on this item by providing additional information not in the ms, or briefly explain why the item is not applicable/relevant for your study

"The program is self-paced, and content is unlocked in a sequential manner. For the first five levels, exercises are unlocked immediately after the prior exercise is complete. For the last four levels, each level corresponds to seven days of being smoke-free. Therefore, within each level, exercises are unlocked immediately after the prior exercise is viewed; however, the next level will not unlock until users record seven consecutive smoke-free days."

5-x) Clarify the level of human involvement

Clarify the level of human involvement (care providers or health professionals, also technical assistance) in the e-intervention or as co-intervention (detail number and expertise of professionals involved, if any, as well as “type of assistance offered, the timing and frequency of the support, how it is initiated, and the medium by which the assistance is delivered”. It may be necessary to distinguish between the level of human involvement required for the trial, and the level of human involvement required for a routine application outside of a RCT setting (discuss under item 21 – generalizability).

	1	2	3	4	5	
subitem not at all important	○	○	◉	○	○	essential

Does your paper address subitem 5-x?

Copy and paste relevant sections from the manuscript (include quotes in quotation marks "like this" to indicate direct quotes from your manuscript), or elaborate on this item by providing additional information not in the ms, or briefly explain why the item is not applicable/relevant for your study

Level of human involvement was minimal, but participants were offered the option to contact study staff with questions: "On the home screen, participants also have options to anonymously send an email to a quit-smoking coach (emails were answered by our study team)."

5-xi) Report any prompts/reminders used

Report any prompts/reminders used: Clarify if there were prompts (letters, emails, phone calls, SMS) to use the application, what triggered them, frequency etc. It may be necessary to distinguish between the level of prompts/reminders required for the trial, and the level of prompts/reminders for a routine application outside of a RCT setting (discuss under item 21 – generalizability).

	1	2	3	4	5	
subitem not at all important	○	○	◉	○	○	essential

Does your paper address subitem 5-xi? *

Copy and paste relevant sections from the manuscript (include quotes in quotation marks "like this" to indicate direct quotes from your manuscript), or elaborate on this item by providing additional information not in the ms, or briefly explain why the item is not applicable/relevant for your study

"All participants were emailed identical once weekly reminders to use their assigned intervention."

5-xii) Describe any co-interventions (incl. training/support)

Describe any co-interventions (incl. training/support): Clearly state any interventions that are provided in addition to the targeted eHealth intervention, as ehealth intervention may not be designed as stand-alone intervention. This includes training sessions and support [1]. It may be necessary to distinguish between the level of training required for the trial, and the level of training for a routine application outside of a RCT setting (discuss under item 21 – generalizability.

	1	2	3	4	5	
subitem not at all important	(●)	( )	( )	( )	( )	essential

Does your paper address subitem 5-xii? *

Copy and paste relevant sections from the manuscript (include quotes in quotation marks "like this" to indicate direct quotes from your manuscript), or elaborate on this item by providing additional information not in the ms, or briefly explain why the item is not applicable/relevant for your study

There were no co-interventions, so this item is not applicable/relevant.

6a) Completely defined pre-specified primary and secondary outcome measures, including how and when they were assessed

Does your paper address CONSORT subitem 6a? *

Copy and paste relevant sections from the manuscript (include quotes in quotation marks "like this" to indicate direct quotes from your manuscript), or elaborate on this item by providing additional information not in the ms, or briefly explain why the item is not applicable/relevant for your study

"For scientific rigor and comparability with other low-intensity behavioral intervention trials [62, 63], the cessation outcome was 30-day point prevalence abstinence (i.e., no smoking at all in the past 30 days). Smoking status was the self-reported response to the question "When was the last time you smoked, or even tried, a cigarette?" We reported abstinence as both complete case and the missing equals smoking imputation. Due to cost and low demand characteristics for false reporting, the SRNT Subcommittee on Biochemical Verification recommends biochemical confirmation is unnecessary in population-based studies with limited face-to-face contact and studies where the optimal data collection methods are through the mail or telephone [64]. Self-reported smoking is a standard method for assessing the efficacy of low-intensity interventions [62, 63]." "Brief process measures, assessed at baseline and the two-month follow-up, were internalized shame (5-item internalized shame subscale of the Social Impact Scale[59]), internalized stigma (9-item internalized stigma subscale of the Lung Cancer Stigma Inventory [36]), depression (10-item CES-D [60]), and generalized anxiety (7-item GAD-7 [61]). Internalized shame refers to the perception that one's illness sets one apart from others who are well, and feeling a need for secrecy about the illness [59]. A sample scale item is: "I feel a need to keep my illness secret." Internalized stigma refers to the internalized experience of rejection, blame, and devaluation based on the assumption that one has caused one's illness [36]. We modified the internalized stigma subscale of the Lung Cancer Stigma Inventory so it focused on cancer broadly (rather than only lung cancer). A sample scale item is: "I blame myself for having cancer."" "Treatment satisfaction outcomes were the extent to which a participant: (1) was overall satisfied with assigned app, (2) would recommend assigned app to friend, and (3) believed assigned app was made for someone like them. Response choices for all items ranged from "Not at all" (1) to "Very much" (5) and were dichotomized such that a threshold of "Somewhat" (3) or higher represented satisfaction."

6a-i) Online questionnaires: describe if they were validated for online use and apply CHERRIES items to describe how the questionnaires were designed/deployed

If outcomes were obtained through online questionnaires, describe if they were validated for online use and apply CHERRIES items to describe how the questionnaires were designed/deployed [9].

	1	2	3	4	5	
subitem not at all important	○	○	○	○	◉	essential

Does your paper address subitem 6a-i?

Copy and paste relevant sections from manuscript text

"All measures were validated for online use from our prior online trials." "Baseline measures. At baseline, participants reported on demographics, their cancer (e.g., type and stage), alcohol use [AUDIT [57]], current and past tobacco use, nicotine dependence [FTND [58]]. confidence in quitting, and smoking in the social environment.
Treatment utilization. Utilization was assessed via data logged automatically by the secured server on how many times the app was opened within the 60 days after randomization. Due to a database error, this data was only available from all 32 participants who were randomized after June 29, 2018.
Treatment satisfaction. Treatment satisfaction outcomes were the extent to which a participant: (1) was overall satisfied with assigned app, (2) would recommend assigned app to friend, and (3) believed assigned app was made for someone like them. Response choices for all items ranged from "Not at all" (1) to "Very much" (5) and were dichotomized such that a threshold of "Somewhat" (3) or higher represented satisfaction.
Process measures. Brief process measures, assessed at baseline and the two-month follow-up, were internalized shame (5-item internalized shame subscale of the Social Impact Scale[59]), internalized stigma (9-item internalized stigma subscale of the Lung Cancer Stigma Inventory [36]), depression (10-item CES-D [60]), and generalized anxiety (7-item GAD-7 [61]). Internalized shame refers to the perception that one's illness sets one apart from others who are well, and feeling a need for secrecy about the illness [59]. A sample scale item is: "I feel a need to keep my illness secret." Internalized stigma refers to the internalized experience of rejection, blame, and devaluation based on the assumption that one has caused one's illness [36]. We modified the internalized stigma subscale of the Lung Cancer Stigma Inventory so it focused on cancer broadly (rather than only lung cancer). A sample scale item is: "I blame myself for having cancer."
Smoking Cessation. For scientific rigor and comparability with other low-intensity behavioral intervention trials [62, 63], the cessation outcome was 30-day point prevalence abstinence (i.e., no smoking at all in the past 30 days). Smoking status was the self-reported response to the question "When was the last time you smoked, or even tried, a cigarette?" We reported abstinence as both complete case and the missing equals smoking imputation. Due to cost and low demand characteristics for false reporting, the SRNT Subcommittee on Biochemical Verification recommends biochemical confirmation is unnecessary in population-based studies with limited face-to-face contact and studies where the optimal data collection methods are through the mail or telephone [64]. Self-reported smoking is a standard method for assessing the efficacy of low-intensity interventions [62, 63]."

6a-ii) Describe whether and how “use” (including intensity of use/dosage) was defined/measured/monitored

Describe whether and how “use” (including intensity of use/dosage) was defined/measured/monitored (logins, logfile analysis, etc.). Use/adoption metrics are important process outcomes that should be reported in any ehealth trial.

	1	2	3	4	5	
subitem not at all important	○	○	○	○	◉	essential

Does your paper address subitem 6a-ii?

Copy and paste relevant sections from manuscript text

"Treatment utilization was assessed via data logged automatically by the secured server on how many times the app was opened within the 60 days after randomization. Due to a database error, this data was only available from all 32 participants who were randomized after June 29, 2018. "

6a-iii) Describe whether, how, and when qualitative feedback from participants was obtained

Describe whether, how, and when qualitative feedback from participants was obtained (e.g., through emails, feedback forms, interviews, focus groups).

	1	2	3	4	5	
subitem not at all important	○	○	◉	○	○	essential

Does your paper address subitem 6a-iii?

Copy and paste relevant sections from manuscript text

"Development of Quit2Heal—smartphone app for smoking cessation among cancer patients. We used an iterative, user-centered design approach [44] to develop an app tailored to help cancer patients quit smoking. Our starting point for the development work was a smartphone app called iCanQuit, which we are currently testing in a large randomized trial for smoking cessation in a general population of adult smokers (NCT02724462). Following the principles of Acceptance and Commitment Therapy (ACT [45]), iCanQuit teaches ACT acceptance skills for coping with cravings and values-focused skills for staying motivated and preventing relapse. To guide the adaptation of iCanQuit for cancer patients who smoke, we interviewed smoking cessation clinicians at four cancer centers across the US and reviewed (1) empirical studies on the psychological factors that influence the likelihood of quitting smoking in cancer patients (i.e., shame, stigma, depression, anxiety [36-38]), (2) the NCCN Clinical Practice Guidelines in Oncology for smoking cessation [18], (3) intervention content from published protocols and trials for cancer patient smoking cessation [43, 46-49], and (4) clinical cancer patient smoking cessation intervention protocols. Subsequently, we conducted in-depth, in-person interviews of six smokers who were currently in treatment for cancer and three caregivers of current cancer patients who smoke. Major themes derived from these interviews were lack of knowledge regarding the effects of smoking on cancer-related outcomes, mental health problems (i.e., depression and anxiety) associated with smoking and cancer, shame about smoking, feeling stigmatized about being a cancer patient who smoked, and fears of seeking support from and/or discomfort discussing smoking and quitting with cancer treatment providers.
Overall, our formative research led us to iteratively develop ACT-framed content on: (1) the consequences of continued smoking vs. quitting smoking for valued-health domains such as daily functioning and cancer treatment outcomes, (2) ACT acceptance skills for coping with the depression and anxiety often associated with a cancer diagnosis, (3) ACT self-compassion exercises for coping with cancer-related stigma and internalized shame, (4) advice on how to seek support for quitting smoking from healthcare providers (e.g., oncologist), and (5) composite testimonials from cancer survivors describing how quitting smoking has allowed them to live more meaningful lives. Wireframes created by our user experience designer were iterated upon in team meetings. They were then user tested with 13 smokers currently receiving cancer treatment to get feedback on usability and content in three iterative rounds of testing. Our user testing also identified cancer patients' choice of the best name for the app, "Quit2Heal." After our developer created an initial beta version of the Quit2Heal app, the study team reviewed it for several weeks to identify edits for the new content and features, as well as any technical bugs.
Our review yielded a second beta version that was tested in a seven-day diary study with five adult smokers (three women, two men) currently receiving cancer treatment who had varying levels of technical ability and confidence in quitting smoking. The diary study included a 30-minute onboarding session, seven nightly 10-minute surveys with closed and open-ended questions about each participant's experience of the app that day, a 10-minute call on day four to discuss their open-ended impressions of the app so far, and a 45-minute exit interview about their overall experience and the usability of the app. All participants rated the app as highly useful overall, were very satisfied overall, and would recommend the app to other cancer patients who smoke. They all liked the five content areas created specifically for cancer patients who smoke. The major problem area was that they were not clear where to start the app's program. Our remedies included: (1) adding an introduction with screen shots showing how to begin the program and (2) greying out the sections that come later in the program until they become available. There were also a few other minor usability concerns that were easily remedied. Following this final round of changes, Quit2Heal was ready for testing in the pilot randomized trial."

6b) Any changes to trial outcomes after the trial commenced, with reasons

Does your paper address CONSORT subitem 6b? *

Copy and paste relevant sections from the manuscript (include quotes in quotation marks "like this" to indicate direct quotes from your manuscript), or elaborate on this item by providing additional information not in the ms, or briefly explain why the item is not applicable/relevant for your study

There were no changes to trial outcomes after the trial commenced, so this item is not applicable/relevant.

7a) How sample size was determined

NPT: When applicable, details of whether and how the clustering by care provides or centers was addressed

7a-i) Describe whether and how expected attrition was taken into account when calculating the sample size

Describe whether and how expected attrition was taken into account when calculating the sample size.

	1	2	3	4	5	
subitem not at all important	○	◉	○	○	○	essential

Does your paper address subitem 7a-i?

Copy and paste relevant sections from manuscript title (include quotes in quotation marks "like this" to indicate direct quotes from your manuscript), or elaborate on this item by providing additional information not in the ms, or briefly explain why the item is not applicable/relevant for your study

Our planned sample size was based on previous pilot trial experience, which accounted for attrition. However, our actual sample size was determined by recruitment costs: "Our original recruitment goal was 200 (100 per arm), based on our experience with recruiting 200 participants in prior pilot randomized trials of mHealth and eHealth of smoking cessation for the general population of smokers [51, 52]. However, by two months into this pilot trial's recruitment period, the Facebook ad algorithms determined that the cost of the Facebook ads to recruit each cancer patient who smokes was 16 times higher than the cost of the Facebook ads to recruit each smoker from the general population (i.e., $213.25 vs. $13.60). Consequently, to meet our limited pilot budget and complete the recruitment within the funding period (12 months), we downward adjusted our recruitment goal to 60."
Attrition was very low: "The 2-month follow-up survey retention rate was 92% (54/59) and did not differ by study arm (P =.15)."

7b) When applicable, explanation of any interim analyses and stopping guidelines

Does your paper address CONSORT subitem 7b? *

Copy and paste relevant sections from the manuscript (include quotes in quotation marks "like this" to indicate direct quotes from your manuscript), or elaborate on this item by providing additional information not in the ms, or briefly explain why the item is not applicable/relevant for your study

There were no interim analyses, and there were no needed stopping guidelines for this pilot trial.

8a) Method used to generate the random allocation sequence

NPT: When applicable, how care providers were allocated to each trial group

Does your paper address CONSORT subitem 8a? *

Copy and paste relevant sections from the manuscript (include quotes in quotation marks "like this" to indicate direct quotes from your manuscript), or elaborate on this item by providing additional information not in the ms, or briefly explain why the item is not applicable/relevant for your study

"Enrolled participants were randomized (1:1) to either the experimental intervention (Quit2Heal, n = 29) or the control intervention (QuitGuide, n = 30). We used randomly permuted block randomization, stratified by Heaviness of Smoking Index (score > 4[53]), confidence in being smoke-free >70 (on a 0 to 100 scale), and recruitment method (i.e., clinic vs. social media)."

8b) Type of randomisation; details of any restriction (such as blocking and block size)

Does your paper address CONSORT subitem 8b? *

Copy and paste relevant sections from the manuscript (include quotes in quotation marks "like this" to indicate direct quotes from your manuscript), or elaborate on this item by providing additional information not in the ms, or briefly explain why the item is not applicable/relevant for your study

"We used computer generated randomly permuted block randomization, stratified by Heaviness of Smoking Index (score > 4[53]), confidence in being smoke-free >70 (on a 0 to 100 scale), and recruitment method (i.e., clinic vs. social media)."

9) Mechanism used to implement the random allocation sequence (such as sequentially numbered containers), describing any steps taken to conceal the sequence until interventions were assigned

Does your paper address CONSORT subitem 9? *

Copy and paste relevant sections from the manuscript (include quotes in quotation marks "like this" to indicate direct quotes from your manuscript), or elaborate on this item by providing additional information not in the ms, or briefly explain why the item is not applicable/relevant for your study

"Randomly permuted block randomization."

10) Who generated the random allocation sequence, who enrolled participants, and who assigned participants to interventions

Does your paper address CONSORT subitem 10? *

Copy and paste relevant sections from the manuscript (include quotes in quotation marks "like this" to indicate direct quotes from your manuscript), or elaborate on this item by providing additional information not in the ms, or briefly explain why the item is not applicable/relevant for your study

"We used computer generated randomly permuted block randomization." "Neither research staff nor study participants had access to upcoming study arm assignments. Study staff and investigators were blind to random assignment throughout the entire duration of the trial."

11a) If done, who was blinded after assignment to interventions (for example, participants, care providers, those assessing outcomes) and how

NPT: Whether or not administering co-interventions were blinded to group assignment

11a-i) Specify who was blinded, and who wasn’t

Specify who was blinded, and who wasn’t. Usually, in web-based trials it is not possible to blind the participants [1, 3] (this should be clearly acknowledged), but it may be possible to blind outcome assessors, those doing data analysis or those administering co-interventions (if any).

	1	2	3	4	5	
subitem not at all important	○	○	○	○	◉	essential

Does your paper address subitem 11a-i? *

Copy and paste relevant sections from the manuscript (include quotes in quotation marks "like this" to indicate direct quotes from your manuscript), or elaborate on this item by providing additional information not in the ms, or briefly explain why the item is not applicable/relevant for your study

"Randomization assignment was concealed from participants throughout the entire trial. Neither research staff nor study participants had access to upcoming randomized study arm assignments. Study staff and investigators were blind to random assignment throughout the entire duration of the trial. "

11a-ii) Discuss e.g., whether participants knew which intervention was the “intervention of interest” and which one was the “comparator”

Informed consent procedures (4a-ii) can create biases and certain expectations - discuss e.g., whether participants knew which intervention was the “intervention of interest” and which one was the “comparator”.

	1	2	3	4	5	
subitem not at all important	○	○	○	○	◉	essential

Does your paper address subitem 11a-ii?

Copy and paste relevant sections from the manuscript (include quotes in quotation marks "like this" to indicate direct quotes from your manuscript), or elaborate on this item by providing additional information not in the ms, or briefly explain why the item is not applicable/relevant for your study

"To ensure participants were blinded to their assigned intervention, each app was branded as “Quit2Heal” and neither mentioned ACT or QuitGuide."

11b) If relevant, description of the similarity of interventions

(this item is usually not relevant for ehealth trials as it refers to similarity of a placebo or sham intervention to a active medication/intervention)

Does your paper address CONSORT subitem 11b? *

Copy and paste relevant sections from the manuscript (include quotes in quotation marks "like this" to indicate direct quotes from your manuscript), or elaborate on this item by providing additional information not in the ms, or briefly explain why the item is not applicable/relevant for your study

This is an e-health trial so this item is not applicable/relevant

12a) Statistical methods used to compare groups for primary and secondary outcomes

NPT: When applicable, details of whether and how the clustering by care providers or centers was addressed

Does your paper address CONSORT subitem 12a? *

Copy and paste relevant sections from the manuscript (include quotes in quotation marks "like this" to indicate direct quotes from your manuscript), or elaborate on this item by providing additional information not in the ms, or briefly explain why the item is not applicable/relevant for your study

"Demographic characteristics, smoking behavior, and process measures at baseline were compared between study groups using two sample t-tests for continuous variables and Fisher's exact test for binary variables. All participants were analyzed in the study arm to which they were randomized.
We used logistic regression models to analyze differences between treatment arms on binary cessation and satisfaction outcomes. A negative binomial model was used to analyze right-skewed app utilization data. Linear models were used to analyze changes in process indicator measures, adjusting for baseline value of the measure. All models were adjusted for the three variables used in stratified randomization. Models were also adjusted for any baseline characteristic that was both imbalanced between study arms at baseline (i.e., P < .10) and associated with the outcome of interest. Statistical tests were two-sided, with α = .05. Analyses were completed using R version 3.6.1 [65] and R library MASS [66]."

12a-i) Imputation techniques to deal with attrition / missing values

Imputation techniques to deal with attrition / missing values: Not all participants will use the intervention/comparator as intended and attrition is typically high in ehealth trials. Specify how participants who did not use the application or dropped out from the trial were treated in the statistical analysis (a complete case analysis is strongly discouraged, and simple imputation techniques such as LOCF may also be problematic [4]).

	1	2	3	4	5	
subitem not at all important	○	○	○	◉	○	essential

Does your paper address subitem 12a-i? *

Copy and paste relevant sections from the manuscript (include quotes in quotation marks "like this" to indicate direct quotes from your manuscript), or elaborate on this item by providing additional information not in the ms, or briefly explain why the item is not applicable/relevant for your study

"Assuming the four participants missing two-month outcome data were smoking (i.e., missing=smoking), the 30-day adjusted point prevalence quit rate was 17% for Quit2Heal vs. 7% for QuitGuide (OR=3.87; 95% CI: 0.57, 26.16; P =.17)."

12b) Methods for additional analyses, such as subgroup analyses and adjusted analyses

Does your paper address CONSORT subitem 12b? *

Copy and paste relevant sections from the manuscript (include quotes in quotation marks "like this" to indicate direct quotes from your manuscript), or elaborate on this item by providing additional information not in the ms, or briefly explain why the item is not applicable/relevant for your study

"All models were adjusted for the three variables used in stratified randomization. Models were also adjusted for any baseline characteristic that was both imbalanced between study arms at baseline (i.e., P < .10) and associated with the outcome of interest." "While none of the measured baseline characteristics significantly differed between study arms (all P>.05), education completed trended toward an imbalance (P = .07) and was predictive of the cessation outcome so we adjusted for this variable in the analyses."

X26) REB/IRB Approval and Ethical Considerations [recommended as subheading under "Methods"] (not a CONSORT item)

X26-i) Comment on ethics committee approval

	1	2	3	4	5	
subitem not at all important	○	○	○	○	◉	essential

Does your paper address subitem X26-i?

Copy and paste relevant sections from the manuscript (include quotes in quotation marks "like this" to indicate direct quotes from your manuscript), or elaborate on this item by providing additional information not in the ms, or briefly explain why the item is not applicable/relevant for your study

"All study activities were reviewed and approved by the Fred Hutchinson Cancer Research Center and Memorial Sloan Kettering Institutional Review Board."

x26-ii) Outline informed consent procedures

Outline informed consent procedures e.g., if consent was obtained offline or online (how? Checkbox, etc.?), and what information was provided (see 4a-ii). See [6] for some items to be included in informed consent documents.

	1	2	3	4	5	
subitem not at all important	○	○	◉	○	○	essential

Does your paper address subitem X26-ii?

Copy and paste relevant sections from the manuscript (include quotes in quotation marks "like this" to indicate direct quotes from your manuscript), or elaborate on this item by providing additional information not in the ms, or briefly explain why the item is not applicable/relevant for your study

Consent was obtained online with a checkbox: "Those who were eligible were instantly sent an email inviting them to provide informed consent and complete the baseline assessment."

X26-iii) Safety and security procedures

Safety and security procedures, incl. privacy considerations, and any steps taken to reduce the likelihood or detection of harm (e.g., education and training, availability of a hotline)

	1	2	3	4	5	
subitem not at all important	○	○	◉	○	○	essential

Does your paper address subitem X26-iii?

Copy and paste relevant sections from the manuscript (include quotes in quotation marks "like this" to indicate direct quotes from your manuscript), or elaborate on this item by providing additional information not in the ms, or briefly explain why the item is not applicable/relevant for your study

"Encrypted online survey" and to access the apps, "password provided only to the study participant."

RESULTS

13a) For each group, the numbers of participants who were randomly assigned, received intended treatment, and were analysed for the primary outcome

NPT: The number of care providers or centers performing the intervention in each group and the number of patients treated by each care provider in each center

Does your paper address CONSORT subitem 13a? *

Copy and paste relevant sections from the manuscript (include quotes in quotation marks "like this" to indicate direct quotes from your manuscript), or elaborate on this item by providing additional information not in the ms, or briefly explain why the item is not applicable/relevant for your study

"Overall, n (%) n=59 QuitGuide, n (%) n=30 Quit2Heal, n (%) n=29"

13b) For each group, losses and exclusions after randomisation, together with reasons

Does your paper address CONSORT subitem 13b? (NOTE: Preferably, this is shown in a CONSORT flow diagram) *

Copy and paste relevant sections from the manuscript (include quotes in quotation marks "like this" to indicate direct quotes from your manuscript), or elaborate on this item by providing additional information not in the ms, or briefly explain why the item is not applicable/relevant for your study

There were no losses and exclusions after randomization, so this item is not applicable/relevant.

13b-i) Attrition diagram

Strongly recommended: An attrition diagram (e.g., proportion of participants still logging in or using the intervention/comparator in each group plotted over time, similar to a survival curve) or other figures or tables demonstrating usage/dose/engagement.

	1	2	3	4	5	
subitem not at all important	○	◉	○	○	○	essential

Does your paper address subitem 13b-i?

Copy and paste relevant sections from the manuscript or cite the figure number if applicable (include quotes in quotation marks "like this" to indicate direct quotes from your manuscript), or elaborate on this item by providing additional information not in the ms, or briefly explain why the item is not applicable/relevant for your study

Since the retention rate was 92% ("2-month follow-up survey retention rate was 92% (54/59) and did not differ by study arm (P =.15)") we did not include an attrition diagram.

14a) Dates defining the periods of recruitment and follow-up

Does your paper address CONSORT subitem 14a? *

Copy and paste relevant sections from the manuscript (include quotes in quotation marks "like this" to indicate direct quotes from your manuscript), or elaborate on this item by providing additional information not in the ms, or briefly explain why the item is not applicable/relevant for your study

"Participants were recruited nationally over a 12-month period from April 2, 2018 to April 1, 2019." "Participants were 59 adult smokers diagnosed with cancer within the past 12 months" and there was "the 2-month trial period".

14a-i) Indicate if critical “secular events” fell into the study period

Indicate if critical “secular events” fell into the study period, e.g., significant changes in Internet resources available or “changes in computer hardware or Internet delivery resources”

	1	2	3	4	5	
subitem not at all important	(●)	( )	( )	( )	( )	essential

Does your paper address subitem 14a-i?

Copy and paste relevant sections from the manuscript (include quotes in quotation marks "like this" to indicate direct quotes from your manuscript), or elaborate on this item by providing additional information not in the ms, or briefly explain why the item is not applicable/relevant for your study

There were no critical “secular events” in the study period so the item is not applicable/relevant.

14b) Why the trial ended or was stopped (early)

Does your paper address CONSORT subitem 14b? *

Copy and paste relevant sections from the manuscript (include quotes in quotation marks "like this" to indicate direct quotes from your manuscript), or elaborate on this item by providing additional information not in the ms, or briefly explain why the item is not applicable/relevant for your study

The trial ended when we met the recruitment goal: "to meet our limited pilot budget and complete the recruitment within the funding period (12 months), we downward adjusted our recruitment goal to 60."

15) A table showing baseline demographic and clinical characteristics for each group

NPT: When applicable, a description of care providers (case volume, qualification, expertise, etc.) and centers (volume) in each group

Does your paper address CONSORT subitem 15? *

Copy and paste relevant sections from the manuscript (include quotes in quotation marks "like this" to indicate direct quotes from your manuscript), or elaborate on this item by providing additional information not in the ms, or briefly explain why the item is not applicable/relevant for your study

A table showing baseline demographic and clinical characteristics for each group is included; see Table 1.

15-i) Report demographics associated with digital divide issues

In ehealth trials it is particularly important to report demographics associated with digital divide issues, such as age, education, gender, social-economic status, computer/Internet/ehealth literacy of the participants, if known.

	1	2	3	4	5	
subitem not at all important	○	○	◉	○	○	essential

Does your paper address subitem 15-i? *

Copy and paste relevant sections from the manuscript (include quotes in quotation marks "like this" to indicate direct quotes from your manuscript), or elaborate on this item by providing additional information not in the ms, or briefly explain why the item is not applicable/relevant for your study

Age, education, gender are included in Table 1. Baseline characteristics

16) For each group, number of participants (denominator) included in each analysis and whether the analysis was by original assigned groups

16-i) Report multiple "denominators" and provide definitions

Report multiple "denominators" and provide definitions: Report N's (and effect sizes) "across a range of study participation [and use] thresholds" [1], e.g., N exposed, N consented, N used more than x times, N used more than y weeks, N participants "used" the intervention/comparator at specific pre-defined time points of interest (in absolute and relative numbers per group). Always clearly define "use" of the intervention.

	1	2	3	4	5	
subitem not at all important	○	○	○	○	◉	essential

Does your paper address subitem 16-i? *

Copy and paste relevant sections from the manuscript (include quotes in quotation marks "like this" to indicate direct quotes from your manuscript), or elaborate on this item by providing additional information not in the ms, or briefly explain why the item is not applicable/relevant for your study

Number of participants in each group is included in Table 2. Primary and secondary study outcomes.

16-ii) Primary analysis should be intent-to-treat

Primary analysis should be intent-to-treat, secondary analyses could include comparing only "users", with the appropriate caveats that this is no longer a randomized sample (see 18-i).

	1	2	3	4	5	
subitem not at all important	○	○	○	○	◉	essential

Does your paper address subitem 16-ii?

Copy and paste relevant sections from the manuscript (include quotes in quotation marks "like this" to indicate direct quotes from your manuscript), or elaborate on this item by providing additional information not in the ms, or briefly explain why the item is not applicable/relevant for your study

"All participants were analyzed intent to treat, in the study arm to which they were randomized."

17a) For each primary and secondary outcome, results for each group, and the estimated effect size and its precision (such as 95% confidence interval)

Does your paper address CONSORT subitem 17a? *

Copy and paste relevant sections from the manuscript (include quotes in quotation marks "like this" to indicate direct quotes from your manuscript), or elaborate on this item by providing additional information not in the ms, or briefly explain why the item is not applicable/relevant for your study

"Participant utilization and satisfaction. As shown in Table 2, compared to QuitGuide participants, Quit2Heal participants: (1) were more satisfied with their assigned app (90% vs. 65%; P =.047), (2) were more likely to report that their assigned app was made for someone like them (86% vs. 62%; P = .04), and (3) opened their app more times during the two-month trial period, although this difference was not statistically significant [M = 10.0 (SD = 14.40) vs. 6.1 (SD =5.3); P =.33].
Smoking outcomes. The self-reported 30-day point prevalence quit rate for those who completed the two-month follow-up was 20% for Quit2Heal vs. 7% for QuitGuide (OR=5.16; 95% CI: 0.71, 37.29; P =.10). Assuming the four participants missing two-month outcome data were smoking (i.e., missing=smoking), the 30-day adjusted point prevalence quit rate was 17% for Quit2Heal vs. 7% for QuitGuide (OR=3.87; 95% CI: 0.57, 26.16; P =.17)."

17a-i) Presentation of process outcomes such as metrics of use and intensity of use

In addition to primary/secondary (clinical) outcomes, the presentation of process outcomes such as metrics of use and intensity of use (dose, exposure) and their operational definitions is critical. This does not only refer to metrics of attrition (13-b) (often a binary variable), but also to more continuous exposure metrics such as “average session length”. These must be accompanied by a technical description how a metric like a “session” is defined (e.g., timeout after idle time) [1] (report under item 6a).

	1	2	3	4	5	
subitem not at all important	○	○	○	●	○	essential

Does your paper address subitem 17a-i?

Copy and paste relevant sections from the manuscript (include quotes in quotation marks "like this" to indicate direct quotes from your manuscript), or elaborate on this item by providing additional information not in the ms, or briefly explain why the item is not applicable/relevant for your study

"Processes of change. From baseline to the two-month follow-up, Quit2Heal participants also reported greater improvement in internalized shame, cancer stigma, depression and anxiety though none of these changes were significant (all P > .05).
Use of outside treatment. Usage of outside treatments to quit smoking during the two-month study period did not differ by study arm: nicotine patch (20% for Quit2Heal vs. 31% for QuitGuide; P = .34), nicotine gum (12% for Quit2Heal vs. 17% for QuitGuide; P = .59), varenicline (16% for Quit2Heal vs. 17% for QuitGuide; P = .89), buproprion (4% for Quit2Heal vs. 7% for QuitGuide; P = .65), any FDA-approved medications for smoking cessation (40% for Quit2Heal vs. 48% for QuitGuide; P = .49), and any other behavioral program (4% for Quit2Heal vs. 0% for QuitGuide; P = .94)."

17b) For binary outcomes, presentation of both absolute and relative effect sizes is recommended

Does your paper address CONSORT subitem 17b? *

Copy and paste relevant sections from the manuscript (include quotes in quotation marks "like this" to indicate direct quotes from your manuscript), or elaborate on this item by providing additional information not in the ms, or briefly explain why the item is not applicable/relevant for your study

"Smoking outcomes. The self-reported 30-day point prevalence quit rate for those who completed the two-month follow-up was 20% for Quit2Heal vs. 7% for QuitGuide (OR=5.16; 95% CI: 0.71, 37.29; P =.10). Assuming the four participants missing two-month outcome data were smoking (i.e., missing=smoking), the 30-day adjusted point prevalence quit rate was 17% for Quit2Heal vs. 7% for QuitGuide (OR=3.87; 95% CI: 0.57, 26.16; P =.17)."

18) Results of any other analyses performed, including subgroup analyses and adjusted analyses, distinguishing pre-specified from exploratory

Does your paper address CONSORT subitem 18? *

Copy and paste relevant sections from the manuscript (include quotes in quotation marks "like this" to indicate direct quotes from your manuscript), or elaborate on this item by providing additional information not in the ms, or briefly explain why the item is not applicable/relevant for your study

None; this item is not applicable/relevant.

18-i) Subgroup analysis of comparing only users

A subgroup analysis of comparing only users is not uncommon in ehealth trials, but if done, it must be stressed that this is a self-selected sample and no longer an unbiased sample from a randomized trial (see 16-iii).

	1	2	3	4	5	
subitem not at all important	○	◉	○	○	○	essential

Does your paper address subitem 18-i?

Copy and paste relevant sections from the manuscript (include quotes in quotation marks "like this" to indicate direct quotes from your manuscript), or elaborate on this item by providing additional information not in the ms, or briefly explain why the item is not applicable/relevant for your study

Due to the small sample size of the pilot, there were no subgroup analyses.

19) All important harms or unintended effects in each group

(for specific guidance see CONSORT for harms)

Does your paper address CONSORT subitem 19? *

Copy and paste relevant sections from the manuscript (include quotes in quotation marks "like this" to indicate direct quotes from your manuscript), or elaborate on this item by providing additional information not in the ms, or briefly explain why the item is not applicable/relevant for your study

No harms or unintended effects were noted in either group in the study.

19-i) Include privacy breaches, technical problems

Include privacy breaches, technical problems. This does not only include physical “harm” to participants, but also incidents such as perceived or real privacy breaches [1], technical problems, and other unexpected/unintended incidents. “Unintended effects” also includes unintended positive effects [2].

	1	2	3	4	5	
subitem not at all important	○	●	○	○	○	essential

Does your paper address subitem 19-i?

Copy and paste relevant sections from the manuscript (include quotes in quotation marks "like this" to indicate direct quotes from your manuscript), or elaborate on this item by providing additional information not in the ms, or briefly explain why the item is not applicable/relevant for your study

There were no privacy breaches or technical problems in the study.

19-ii) Include qualitative feedback from participants or observations from staff/researchers

Include qualitative feedback from participants or observations from staff/researchers, if available, on strengths and shortcomings of the application, especially if they point to unintended/unexpected effects or uses. This includes (if available) reasons for why people did or did not use the application as intended by the developers.

	1	2	3	4	5	
subitem not at all important	●	○	○	○	○	essential

Does your paper address subitem 19-ii?

Copy and paste relevant sections from the manuscript (include quotes in quotation marks "like this" to indicate direct quotes from your manuscript), or elaborate on this item by providing additional information not in the ms, or briefly explain why the item is not applicable/relevant for your study

The study did not collect qualitative feedback from participants or observations from staff/researchers.

DISCUSSION

22) Interpretation consistent with results, balancing benefits and harms, and considering other relevant evidence

NPT: In addition, take into account the choice of the comparator, lack of or partial blinding, and unequal expertise of care providers or centers in each group

22-i) Restate study questions and summarize the answers suggested by the data, starting with primary outcomes and process outcomes (use)

Restate study questions and summarize the answers suggested by the data, starting with primary outcomes and process outcomes (use).

	1	2	3	4	5	
subitem not at all important	○	○	○	○	◉	essential

Does your paper address subitem 22-i? *

Copy and paste relevant sections from the manuscript (include quotes in quotation marks "like this" to indicate direct quotes from your manuscript), or elaborate on this item by providing additional information not in the ms, or briefly explain why the item is not applicable/relevant for your study

"This paper reports trial recruitment, retention, participant acceptability, preliminary effectiveness, and impact on processes of change of the first-known smartphone app for smoking cessation tailored for cancer patients. In general, the results supported all the pilot study aims."

22-ii) Highlight unanswered new questions, suggest future research

Highlight unanswered new questions, suggest future research.

	1	2	3	4	5	
subitem not at all important	○	○	○	◉	○	essential

Does your paper address subitem 22-ii?

Copy and paste relevant sections from the manuscript (include quotes in quotation marks "like this" to indicate direct quotes from your manuscript), or elaborate on this item by providing additional information not in the ms, or briefly explain why the item is not applicable/relevant for your study

"The study results suggest these main lines of future research: (1) provide a definitive test of the effectiveness for smoking cessation of smartphone-delivered Quit2Heal compared with QuitGuide—an app that follows US Clinical Practice Guidelines; (2) demonstrate that the smoking cessation outcomes of Quit2Heal, but not QuitGuide, are mediated by processes that impede cancer patients’ cessation (i.e., internalized shame, cancer stigma, depression, and anxiety), and (3) explore reasons for refusal and baseline moderators of treatment effectiveness."

20) Trial limitations, addressing sources of potential bias, imprecision, and, if relevant, multiplicity of analyses

20-i) Typical limitations in ehealth trials

Typical limitations in ehealth trials: Participants in ehealth trials are rarely blinded. Ehealth trials often look at a multiplicity of outcomes, increasing risk for a Type I error. Discuss biases due to non-use of the intervention/usability issues, biases through informed consent procedures, unexpected events.

	1	2	3	4	5	
subitem not at all important	○	○	○	◉	○	essential

Does your paper address subitem 20-i? *

Copy and paste relevant sections from the manuscript (include quotes in quotation marks "like this" to indicate direct quotes from your manuscript), or elaborate on this item by providing additional information not in the ms, or briefly explain why the item is not applicable/relevant for your study

Participants were blinded, but there were other limitations: "The study has several important limitations. As a pilot randomized trial, the study's sample size was not powered to detect statistically significant differences in quit rates or to conduct formal moderation or mediation analysis of hypothesized treatment effects. Moreover, there is substantial smoking relapse that naturally occurs after a two-month follow-up [63, 69], especially among cancer patients who smoke, and therefore a longer-term follow-up (e.g., 12 months) is recommended. Due to a technical error, automatic recording of utilization data did not occur until two months after the beginning of the trial recruitment period. Finally, we relied exclusively on self-reported abstinence in our estimate of 30-day point prevalence abstinence."

21) Generalisability (external validity, applicability) of the trial findings

NPT: External validity of the trial findings according to the intervention, comparators, patients, and care providers or centers involved in the trial

21-i) Generalizability to other populations

Generalizability to other populations: In particular, discuss generalizability to a general Internet population, outside of a RCT setting, and general patient population, including applicability of the study results for other organizations

	1	2	3	4	5	
subitem not at all important	○	○	◉	○	○	essential

Does your paper address subitem 21-i?

Copy and paste relevant sections from the manuscript (include quotes in quotation marks "like this" to indicate direct quotes from your manuscript), or elaborate on this item by providing additional information not in the ms, or briefly explain why the item is not applicable/relevant for your study

As a pilot trial, this was not addressed.

21-ii) Discuss if there were elements in the RCT that would be different in a routine application setting

Discuss if there were elements in the RCT that would be different in a routine application setting (e.g., prompts/reminders, more human involvement, training sessions or other co-interventions) and what impact the omission of these elements could have on use, adoption, or outcomes if the intervention is applied outside of a RCT setting.

	1	2	3	4	5	
subitem not at all important	○	○	◉	○	○	essential

Does your paper address subitem 21-ii?

Copy and paste relevant sections from the manuscript (include quotes in quotation marks "like this" to indicate direct quotes from your manuscript), or elaborate on this item by providing additional information not in the ms, or briefly explain why the item is not applicable/relevant for your study

Not different from routine setting.

OTHER INFORMATION

23) Registration number and name of trial registry

Does your paper address CONSORT subitem 23? *

Copy and paste relevant sections from the manuscript (include quotes in quotation marks "like this" to indicate direct quotes from your manuscript), or elaborate on this item by providing additional information not in the ms, or briefly explain why the item is not applicable/relevant for your study

"Clinical Trials.gov Registration Number: NCT03600038"

24) Where the full trial protocol can be accessed, if available

Does your paper address CONSORT subitem 24? *

Cite a Multimedia Appendix, other reference, or copy and paste relevant sections from the manuscript (include quotes in quotation marks "like this" to indicate direct quotes from your manuscript), or elaborate on this item by providing additional information not in the ms, or briefly explain why the item is not applicable/relevant for your study

The full protocol can be accessed at ClinicalTrials.gov:
https://clinicaltrials.gov/ct2/show/NCT03600038

25) Sources of funding and other support (such as supply of drugs), role of funders

Does your paper address CONSORT subitem 25? *

Copy and paste relevant sections from the manuscript (include quotes in quotation marks "like this" to indicate direct quotes from your manuscript), or elaborate on this item by providing additional information not in the ms, or briefly explain why the item is not applicable/relevant for your study

"CVS Health Foundation, National Cancer Institute (R01 CA166646; R01CA192849); National Institute on Drug Abuse (R01 DA038411). Funders had no role in the study."

X27) Conflicts of Interest (not a CONSORT item)

X27-i) State the relation of the study team towards the system being evaluated

In addition to the usual declaration of interests (financial or otherwise), also state the relation of the study team towards the system being evaluated, i.e., state if the authors/evaluators are distinct from or identical with the developers/sponsors of the intervention.

	1	2	3	4	5	
subitem not at all important	○	○	●	○	○	essential

Does your paper address subitem X27-i?

Copy and paste relevant sections from the manuscript (include quotes in quotation marks "like this" to indicate direct quotes from your manuscript), or elaborate on this item by providing additional information not in the ms, or briefly explain why the item is not applicable/relevant for your study

The paper makes the following disclosure: "Dr. Jonathan Bricker serves on the Scientific Advisory Board of Chrono Therapeutics. Dr. Heffner has received research support from Pfizer. Other authors have no declarations." There were no conflicts of interest.

About the CONSORT EHEALTH checklist

As a result of using this checklist, did you make changes in your manuscript? *

- ○ yes, major changes
- ◉ yes, minor changes
- ○ no

What were the most important changes you made as a result of using this checklist?

None.

How much time did you spend on going through the checklist INCLUDING making changes in your manuscript *

8 hours on the checklist

As a result of using this checklist, do you think your manuscript has improved? *

- ○ yes
- ◉ no
- ○ Other:

Would you like to become involved in the CONSORT EHEALTH group?

This would involve for example becoming involved in participating in a workshop and writing an "Explanation and Elaboration" document

- ○ yes
- ○ no
- ○ Other:

Any other comments or questions on CONSORT EHEALTH

Your answer

STOP - Save this form as PDF before you click submit

To generate a record that you filled in this form, we recommend to generate a PDF of this page (on a Mac, simply select "print" and then select "print as PDF") before you submit it.

When you submit your (revised) paper to JMIR, please upload the PDF as supplementary file.

Don't worry if some text in the textboxes is cut off, as we still have the complete information in our database. Thank you!

Final step: Click submit !

Click submit so we have your answers in our database!

SUBMIT

Never submit passwords through Google Forms.

This content is neither created nor endorsed by Google. Report Abuse - Terms of Service - Privacy Policy

Google Forms
